# Tumor Genomic Biomarkers as Prognostic Modifiers of Outcomes Following CD19 CAR T-Cell Therapy in Aggressive Large B-Cell Lymphoma: A Systematic Review and Exploratory Meta-Analysis

**DOI:** 10.3390/genes17070752

**Published:** 2026-06-30

**Authors:** Jingke Yang, Heather Hatcher, Harshad Kulkarni, Chris A. Learn

**Affiliations:** Parexel International, LLC., Raleigh, NC 27609, USA; jingke.yang@parexel.com (J.Y.); heather.hatcher@parexel.com (H.H.); harshad.kulkarni@parexel.com (H.K.)

**Keywords:** CD19 CAR T-cell therapy, aggressive large B-cell lymphoma, TP53, double-hit lymphoma, cell of origin, tumor genomic biomarkers, prognosis, systematic review, meta-analysis

## Abstract

**Background/Objectives**: Outcomes after CD19-directed chimeric antigen receptor (CAR) T-cell therapy for relapsed or refractory (R/R) aggressive large B-cell lymphoma (aLBCL) remain heterogeneous. Tumor genomic biomarkers, such as TP53 alteration, MYC/BCL2/BCL6 rearrangement-defined double-hit or triple-hit lymphoma (DHL/THL), cell of origin (COO), and complex karyotype, are established or candidate prognostic factors in conventionally treated lymphoma, but their relevance after CAR T-cell therapy is uncertain. We conducted a systematic review with exploratory meta-analysis of biomarker-stratified outcomes after CD19 CAR T-cell therapy in aLBCL. **Methods**: We searched MEDLINE, Embase, and Web of Science/BIOSIS (April 2026), with targeted PubMed citation lookup during full-text retrieval (PROSPERO CRD420261350514). Eligible studies enrolled adults with R/R disease treated with protocol-eligible CD19 CAR T-cell therapy and reported prespecified tumor genomic biomarkers with stratified outcomes. Random-effects models, using restricted maximum-likelihood estimation with Hartung–Knapp–Sidik–Jonkman (HKSJ) adjustment, were fitted when at least three comparable, non-overlapping studies provided extractable data. **Results**: After duplicate removal, 182 records were screened, 37 were assessed for eligibility, and 26 studies were included in the qualitative synthesis; 10 contributed to 4 pooled analyses. DHL/THL-positive disease was associated with worse unadjusted overall survival (OS) (hazard ratio [HR] 1.52; 95% confidence interval [CI], 1.21–1.89; 95% prediction interval (PI), 0.56–4.08), and non-Germinal center B-cell-like (GCB)/ABC COO with worse adjusted progression-free survival (PFS) (HR 1.44; 95% CI, 1.04–2.00; 95% PI, 0.86–2.43). The complete-response analyses for TP53 alteration (OR 1.30; 95% CI, 0.01–156.60) and COO (OR 1.27; 95% CI, 0.24–6.61) were statistically uninformative. No study permitted evaluation of complex karyotypes. **Conclusions**: Biomarker-stratified evidence after CD19 CAR T-cell therapy is sparse and inconsistently reported. DHL/THL status and non-GCB/activated B-cell-like (ABC) COO showed exploratory survival signals, whereas the TP53 and COO complete-response analyses were uninformative. These biomarkers remain hypothesis-generating rather than validated predictors of CAR T-cell outcome, and standardized, prospective biomarker-stratified reporting is needed.

## 1. Introduction

Relapsed or refractory (R/R) aggressive B-cell lymphomas (aBCL) encompass a biologically and clinically heterogeneous group of lymphoid malignancies, including diffuse large B-cell lymphoma (DLBCL), high-grade B-cell lymphoma (HGBCL), transformed follicular lymphoma, and primary mediastinal B-cell lymphoma (PMBCL) [1,2]. DLBCL is the most common aggressive non-Hodgkin lymphoma. Although frontline chemoimmunotherapy with rituximab, cyclophosphamide, doxorubicin, vincristine, and prednisone (R-CHOP) cures a majority of patients, approximately a third develop refractory disease or relapse [3,4]. Before the advent of cellular therapy, outcomes for patients with refractory or early-relapsing disease were poor, particularly for those who could not achieve durable disease control with platinum-based salvage therapy and consolidative autologous stem-cell transplantation [5].

CD19-directed chimeric antigen receptor (CAR) T-cell therapy has substantially changed this landscape. Three commercially available CD19 CAR T-cell products—axicabtagene ciloleucel, tisagenlecleucel, and lisocabtagene maraleucel—produce durable responses in a subset of patients who previously had limited therapeutic options, and two are now approved in the second-line setting for high-risk disease [6,7,8,9,10]. Outcomes after CAR T-cell therapy nevertheless remain heterogeneous: some patients achieve durable complete remission, whereas others experience primary resistance, early progression, or relapse after an initial response [6,7,8,11]. Immune effector cell toxicities, principally cytokine release syndrome (CRS) and immune effector cell-associated neurotoxicity syndrome (ICANS), also vary across patients, products, and treatment settings [9,12]. A clearer understanding of the biologic determinants of response, resistance, and durability after CAR T-cell therapy is therefore an important unmet need.

Tumor genomic biomarkers are central candidates for this purpose. TP53 alteration, MYC with BCL2 and/or BCL6 rearrangement-defined double-hit or triple-hit lymphoma (DHL/THL), and COO classification are well-established adverse prognostic factors in aBCL treated with conventional chemoimmunotherapy [13,14,15,16]. Complex karyotype has likewise been associated with inferior outcomes in several B-cell malignancies [17,18]. Whether these biomarkers retain the same prognostic weight after CD19 CAR T-cell therapy is less certain. It remains unclear whether their adverse effect persists, is attenuated by the distinct mechanism of cellular therapy, or varies across CAR T-cell products, lines of therapy, and treatment settings. Reported biomarker-stratified outcomes in CAR T-cell cohorts have been variable and at times conflicting, with individual studies frequently constrained by small sample size, incomplete biomarker testing, inconsistent outcome definitions, and overlapping patient populations.

Existing reviews of CAR T-cell therapy in R/R aLBCL have largely addressed product-level efficacy, safety, and real-world effectiveness rather than systematically evaluating tumor genomic biomarkers as prognostic modifiers of response and survival. Consequently, clinicians and trialists lack a consolidated evidence map identifying which biomarker–outcome associations are currently quantifiable, which remain limited to narrative evidence, and where standardized biomarker-stratified reporting is still required.

To address this gap, we conducted a systematic review with exploratory meta-analysis of the association between prespecified tumor genomic biomarkers and outcomes after CD19-directed CAR T-cell therapy in adults with R/R aLBCL. The review prespecified four biomarker categories—TP53 alteration, DHL/THL status, COO classification, and complex karyotype—and evaluated biomarker-stratified complete response (CR), PFS, OS, and immune effector cell toxicities, pooling effect estimates where a sufficient number of comparable, non-overlapping studies provided extractable data and summarizing the remaining evidence narratively.

## 2. Materials and Methods

### 2.1. Search Strategy

This systematic review was conducted according to the guidelines of the Preferred Reporting Items for Systematic Reviews and Meta-Analyses (PRISMA) 2020 statement [19]. The protocol for this review was registered prospectively in a public database (CRD420261350514, dated 25 March 2026). Complete search strategies, search dates, database platforms, platform-specific syntax, filters, limits, and record accounting are provided in Supplementary Material S1.

Systematic database searches were conducted in April-May 2026 in MEDLINE and Embase through ProQuest Dialog and in Web of Science Core Collection with BIOSIS Previews through Clarivate. The search strategy combined terms for three core inclusion concepts: aggressive large B-cell lymphoma, CD19-directed CAR T-cell therapy, and prespecified tumor genomic biomarkers, including TP53 alteration, MYC/BCL2/BCL6 rearrangement-defined double-hit or triple-hit lymphoma, cell of origin, and complex karyotype. Controlled vocabulary terms were used where available, including MeSH terms for MEDLINE and Emtree terms for Embase, together with free-text terms adapted to each platform.

The Web of Science/BIOSIS search used one main Topic-field search covering all biomarker classes and four focused supplemental searches for TP53, DHL/THL, cell of origin, and complex karyotype. PubMed was used only for targeted citation lookup during full-text retrieval and bibliographic verification, not as a separate comprehensive database search; two PubMed records were added to confirm superseding full-text reports identified during screening or citation chasing. No language restrictions were applied at the search step or during screening.

**Figure 1:**
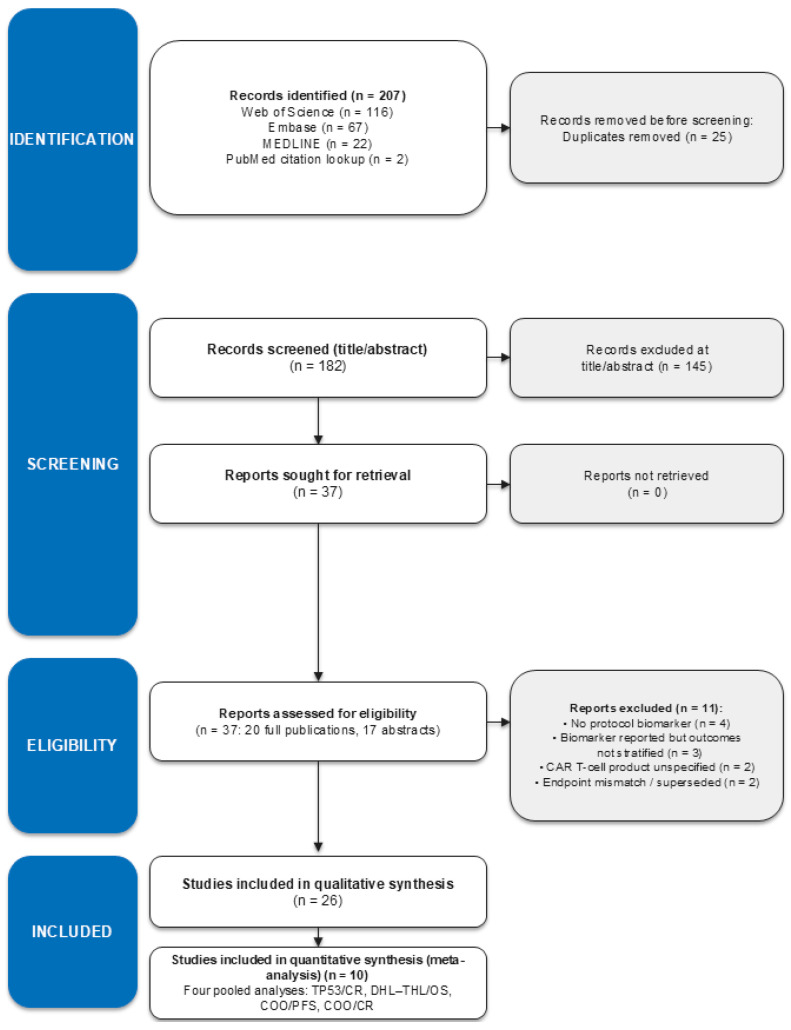
PRISMA 2020 flow diagram. Records were identified from systematic database searches (Web of Science/BIOSIS, n = 116; Embase, n = 67; MEDLINE, n = 22) and targeted PubMed citation lookup for superseding full-text reports (n = 2), yielding 207 records. After deduplication, 182 records were screened by title and abstract, 37 reports were assessed for eligibility, and 26 studies were included in the qualitative synthesis. Ten studies contributed to the quantitative synthesis across four pooled analyses. CAR T, chimeric antigen receptor T-cell therapy.

Systematic database searches yielded 205 records: Web of Science/BIOSIS (n = 116), Embase (n = 67), and MEDLINE (n = 22). With the two targeted PubMed citation-lookup records, 207 records entered cross-source deduplication. After removal of 25 duplicate records, 182 unique records proceeded to title and abstract screening. The PRISMA flow is shown in Figure 1, and PRISMA checklist and eligibility-support materials are provided in Supplementary Material S2.

### 2.2. Eligibility Criteria

Studies were eligible if they met all of the following criteria: (1) prospective clinical trial, registry, retrospective cohort, or mixed retrospective–prospective observational design; (2) adult patients with R/R aLBCL, including DLBCL, HGBCL, transformed follicular lymphoma, or PMBCL; (3) treatment with protocol-eligible single-target CD19-directed CAR T-cell therapy; (4) reporting of at least one prespecified tumor genomic biomarker subgroup, including TP53 alteration, MYC/BCL2/BCL6 rearrangement-defined DHL/THL, COO, or complex karyotype; and (5) reporting of at least one clinical outcome stratified by biomarker status, or sufficient data to derive a biomarker-stratified effect estimate. Eligible comparisons contrasted biomarker-positive groups with the corresponding biomarker-negative group within the same CAR T-cell-treated cohort.

Reports were excluded if they were case reports, very small non-comparative case series, reviews, editorials, commentaries, protocol-only records, pediatric-only studies, non-human or preclinical studies, or purely mechanistic studies without relevant clinical outcome data. Studies of mixed lymphoma populations were excluded unless aLBCL data were separately extractable. Studies of non-CD19 CAR T-cell therapy, nonstandard or unapproved cellular therapy platforms, repeat or multiple CAR T-cell infusions, or CD19 CAR T-cell therapy given concurrently with other targeted or cellular therapies were excluded unless eligible CD19 CAR T-cell data could be separately extracted.

Conference abstracts were eligible if they provided sufficient extractable biomarker-stratified clinical data and represented a unique cohort. When multiple reports described overlapping or linked cohorts, the most complete or most mature report for a given endpoint was retained for primary synthesis. Related reports from the same cohort were used only for eligibility clarification, endpoint-specific extraction, or sensitivity analyses when appropriate.

### 2.3. Study Selection and Data Handling

Two reviewers (J.Y. and H.H.) independently screened titles and abstracts against the prespecified eligibility criteria. Reports judged potentially eligible by either reviewer proceeded to eligibility assessment. Disagreements were resolved by discussion and, when needed, consultation with a third reviewer (C.A.L.).

Search records were exported in RIS and spreadsheet formats and deduplicated using PMID, DOI, title, and author/year matching. Screening decisions, eligibility decisions, and exclusion reasons were recorded in a structured screening log. When multiple reports described overlapping or linked cohorts, the most complete or most mature report for the relevant endpoint was retained for primary synthesis; related reports from the same cohort were used only for eligibility clarification, endpoint-specific extraction, or sensitivity analyses when appropriate.

### 2.4. PRISMA Summary

Record flow was documented according to PRISMA 2020. Records were tracked through identification, deduplication, title and abstract screening, report retrieval, eligibility assessment, qualitative synthesis, and quantitative synthesis. Reasons for exclusion at the eligibility stage were recorded according to the prespecified eligibility criteria. The PRISMA flow diagram is presented in Figure 1, and the PRISMA checklist, eligibility-assessed reports, and excluded reports with primary exclusion reasons are provided in Supplementary Material S2.

### 2.5. Data Extraction and Quality Assessment

Data extraction was performed independently by two reviewers (J.Y. and H.H.) using a standardized, electronic extraction form based on the prespecified PICO framework. Discrepancies were resolved by discussion and, when necessary, consultation with a third reviewer (C.A.L.).

Extracted variables included study identification, publication type, geography, study design and setting, CAR T-cell product, line of therapy, cohort size, biomarker-evaluable denominators, biomarker assay method, biomarker definition, biomarker prevalence, and clinical outcomes stratified by biomarker status. Prespecified outcomes included CR, overall response rate (ORR), PFS, OS, duration of response (DOR), grade ≥ 3 CRS, grade ≥ 3 ICANS, and treatment-related mortality (TRM), where reported. For time-to-event outcomes, adjusted and unadjusted hazard ratios (HRs) with 95% confidence intervals (CIs) were extracted separately. For binary response outcomes, biomarker-stratified 2 × 2 data were extracted when available. Full study-level characteristics, biomarker definitions, and extracted outcomes are provided in Supplementary Tables S3.1–S3.3.

Direction of effect conventions were harmonized before synthesis. The biomarker-positive group was defined as TP53-altered or TP53-mutant disease, DHL/THL-positive disease, or non-GCB/ABC COO; the corresponding comparator groups were TP53 wild-type, non-DHL/THL, and GCB, respectively. For CR and ORR analyses, the event was response; therefore, OR > 1 indicated higher odds of response in the biomarker-positive group. Event-free survival was not pooled with PFS because of endpoint non-comparability; such estimates were retained for evidence mapping or narrative summary only.

Risk of bias was assessed independently by two reviewers using the Quality in Prognosis Studies (QUIPS) tool [20]. Overall study-level risk of bias was classified as low, moderate, or high, considering the QUIPS domains of study participation, study attrition, prognostic factor measurement, outcome measurement, study confounding, and statistical analysis and reporting. Disagreements were resolved by discussion and, when needed, by consultation with the third reviewer. Overall QUIPS ratings and concise rationales are provided in Supplementary Table S3.4.

### 2.6. Data Synthesis

Quantitative synthesis was performed in May 2026 when at least three non-overlapping studies provided comparable, extractable effect estimates or complete-response data for the same biomarker–endpoint comparison. Time to events were pooled as hazard ratios (HRs), and Binary complete-response outcomes were pooled as odds ratio (OR). Random-effects models were fitted using restricted maximum-likelihood estimation of the between-study variance (τ^2^), with Hartung–Knapp–Sidik–Jonkman (HKSJ) confidence-interval correction for the pooled estimate [21]. Analyses were conducted in R using the meta package [22]; detailed model outputs are provided in Supplementary Material S5. This k ≥ 3 threshold was prespecified as a pragmatic minimum to avoid two-study primary meta-analyses, for which estimation of between-study variance and random-effects inference is particularly unstable; even the resulting three-study pools were treated as exploratory.

For response analyses, the event was achieving CR; therefore, OR > 1 indicated higher odds of CR in the biomarker-positive group. For survival analyses, HR > 1 indicated worse survival in the biomarker-positive group. For COO analyses, non-GCB/ABC was treated as the biomarker-positive group and GCB as the comparator.

Between-study heterogeneity was assessed using I^2^, τ^2^, and Cochran’s Q statistic [23]. PIs were reported for HR-based pools when interpretable. PIs were not shown for the CR pools because each binary-outcome pool included only three studies with sparse event data. Prespecified biomarker–endpoint combinations that did not meet the k ≥ 3 threshold were not pooled; extracted study-level data are presented in Supplementary Material S3, and the pooling feasibility assessment is provided in Supplementary Material S4.

Sensitivity analyses included leave-one-out influence analyses and cohort-exclusion analyses for studies flagged for potential overlap or linkage. Because each primary pooled analysis included three studies, all sensitivity estimates after omission of one study or flagged cohort were based on two-study models and were interpreted descriptively. For consistency, sensitivity analyses were re-fitted using the same model specification as the corresponding primary analyses [22,23]. Low-risk-only sensitivity analysis was not feasible because no included study was rated low risk. Prespecified subgroup analyses by CAR T-cell product, costimulatory domain, line of therapy, and study setting were not feasible because each pooled analysis included only three studies. Meta-regression and funnel-plot or regression-based publication-bias assessments were not performed because no pooled analysis approached the prespecified k ≥ 10 threshold [24].

## 3. Results

### 3.1. Study Selection and PRISMA Flow

The flow of records through the review is summarized in Figure 1. Systematic searches of Web of Science Core Collection with BIOSIS Previews (n = 116), Embase (n = 67), and MEDLINE (n = 22) identified 205 records; a further 2 records were obtained through targeted PubMed citation lookup to confirm superseding full-text reports identified during screening or bibliographic verification, giving 207 records in total. After removal of 25 duplicates, 182 unique records underwent title and abstract screening, of which 145 were excluded.

The remaining 37 reports—20 full-text publications or research letters and 17 conference or meeting abstracts—were assessed for eligibility. Eleven reports were excluded because no protocol-specified tumor genomic biomarker was analyzed (n = 4), biomarker status was reported but outcomes were not stratified by biomarker status (n = 3), the CAR T-cell product was unspecified or did not meet the protocol definition of CD19-directed CAR T-cell therapy (n = 2), or the report had an endpoint mismatch or was superseded by a more complete report of the same cohort (n = 2). Details of the excluded reports are provided in Supplementary Table S2.3.

Twenty-six studies were included in the qualitative synthesis and listed in Supplementary Table S3.1. These studies contributed evidence across three of the four prespecified tumor genomic biomarker categories: TP53 alteration (k = 11 biomarker-specific reports), MYC plus BCL2 and/or BCL6 rearrangement-defined double-hit or triple-hit lymphoma (DHL/THL; k = 12 biomarker-specific reports), and cell of origin (COO; k = 10 biomarker-specific reports). The categories were not mutually exclusive, as several studies contributed data to more than one biomarker category. No eligible study provided extractable complex-karyotype-stratified CD19 CAR T-cell outcome data; therefore, no complex-karyotype synthesis was performed. Full per-study characteristics are provided in Supplementary Table S3.1.

Of the 26 included studies, 10 provided extractable biomarker-stratified data suitable for quantitative synthesis across four prespecified pooled analyses of CR and survival outcomes by TP53 alteration, DHL/THL status, and COO (Table 1). The remaining biomarker–endpoint combinations were summarized narratively because fewer than three comparable, non-overlapping studies provided extractable effect estimates or complete-response data. The feasibility assessment for each prespecified analysis is provided in Supplementary Material S4.

### 3.2. Study Characteristics

The 26 studies included in the qualitative synthesis were heterogeneous with respect to publication format, geography, study setting, CAR T-cell product, line of therapy, and biomarker assessment (Table 2; Supplementary Table S3.1). The evidence base comprised 17 full-text articles or research letters and 9 conference abstracts. Most were retrospective real-world cohorts or registry-based analyses, including one electronic health record (EHR)-derived dataset; a smaller number were trial-derived (subgroup or exploratory biomarker analyses of phase 1–3 trials). Cohorts were reported from North America, Europe, and Asia, and included multinational datasets.

Commercial CD19-directed CAR T-cell products were represented, most commonly axicabtagene ciloleucel, tisagenlecleucel, and lisocabtagene maraleucel; several studies used mixed or not-reported products, and China-based cohorts also included relmacabtagene autoleucel or other CD19 CAR T-cell products. Most studies evaluated third-line or later R/R aLBCL, with fewer in second-line or mixed-line settings. Reported outcomes included CR or ORR, PFS or EFS, OS, DOR, CRS and ICANS, and TRM. Biomarker definitions, assay methods, endpoint definitions, and adjustment approaches varied across studies, limiting direct comparability; these details are summarized in Supplementary Tables S3.2 and S3.3.

### 3.3. Risk of Bias Assessment

Risk of bias was assessed using the Quality in Prognosis Studies (QUIPS) framework [20]. Overall ratings are summarized in Supplementary Table S3.4. Among the 26 studies included in the qualitative synthesis, overall risk of bias was moderate or high; no study was classified as low risk. Thirteen studies were rated as moderate risk and thirteen as high risk.

High-risk ratings were most commonly assigned to conference abstracts, small cohorts, studies with incomplete biomarker–outcome reporting, studies lacking extractable hazard ratios or 2 × 2 response data, and studies with limited adjustment for confounding. Common limitations across the evidence base included retrospective design, heterogeneous biomarker definitions, variable endpoint reporting, and incomplete adjustment for clinically relevant prognostic factors.

A low-risk-only sensitivity analysis was not feasible because no pooled analysis retained at least three low-risk studies. Risk-of-bias findings were therefore incorporated through cautious interpretation of the exploratory pooled estimates rather than through formal risk-of-bias-restricted meta-analysis.

### 3.4. TP53 Alterations

TP53 alteration was represented in 11 biomarker-specific reports in the qualitative synthesis: Gao 2023 [35], Porpaczy 2021 [36], Shouval 2022 [25], Shi 2023 [37], Xue 2024 [38], Phuoc 2021 [26], Liu 2025a [27], Sworder 2023 [39], Batlevi 2022 [40], Liu 2025b [41]. Biomarker definitions and assay methods varied across studies and included targeted next-generation sequencing, broader sequencing-based genomic profiling, copy-number assessment, and circulating tumor DNA approaches (Supplementary Table S3.2-A). Survival endpoints were sparsely and inconsistently reported; therefore, the prespecified TP53 survival analyses for PFS and OS were not poolable. Only the complete-response analysis met the prespecified threshold of at least three comparable, non-overlapping studies.

#### 3.4.1. Complete Response

Three studies contributed extractable complete-response data by TP53 status: Shouval 2022 [25], Phuoc 2021 [26], and Liu 2025a [27]. The extracted complete-response data were 10/29 vs. 33/51 for Shouval 2022 [25], 5/7 vs. 1/8 for Phuoc 2021 [26], and 21/53 vs. 37/99 for Liu 2025a [27], comparing TP53-altered or TP53-mutant patients with TP53 wild-type patients. In the random-effects meta-analysis, TP53 alteration was not clearly associated with complete response: pooled OR 1.30 (95% CI, 0.01–156.60), with substantial heterogeneity (I^2^ = 80.8%; τ^2^ = 2.68; Q = 10.4, *p* = 0.006) (Table 1; Supplementary Material S5).

The study-level estimates were discordant. Shouval 2022 [25] showed lower odds of complete response in TP53-altered patients (OR 0.29; 95% CI, 0.11–0.75), Liu 2025a [27] showed a near-null estimate (OR 1.10; 95% CI, 0.55–2.18), and Phuoc 2021 [26] showed a large opposite-direction estimate (OR 17.50; 95% CI, 1.22–250.37) based on a small 15-patient cohort with sparse cells. Leave-one-out analysis confirmed that the pooled TP53 complete-response estimate was unstable. Omission of Phuoc 2021 [26] shifted the two-study point estimate below 1.0, whereas omission of either of the other contributing studies produced highly imprecise estimates. Because each sensitivity analysis included only two studies, these results were interpreted descriptively and did not provide a reliable basis for inference (Supplementary Material S6). Accordingly, the TP53 complete-response pool was considered inconclusive rather than evidence of a consistent association.

#### 3.4.2. Survival Outcomes

**Figure 2:**
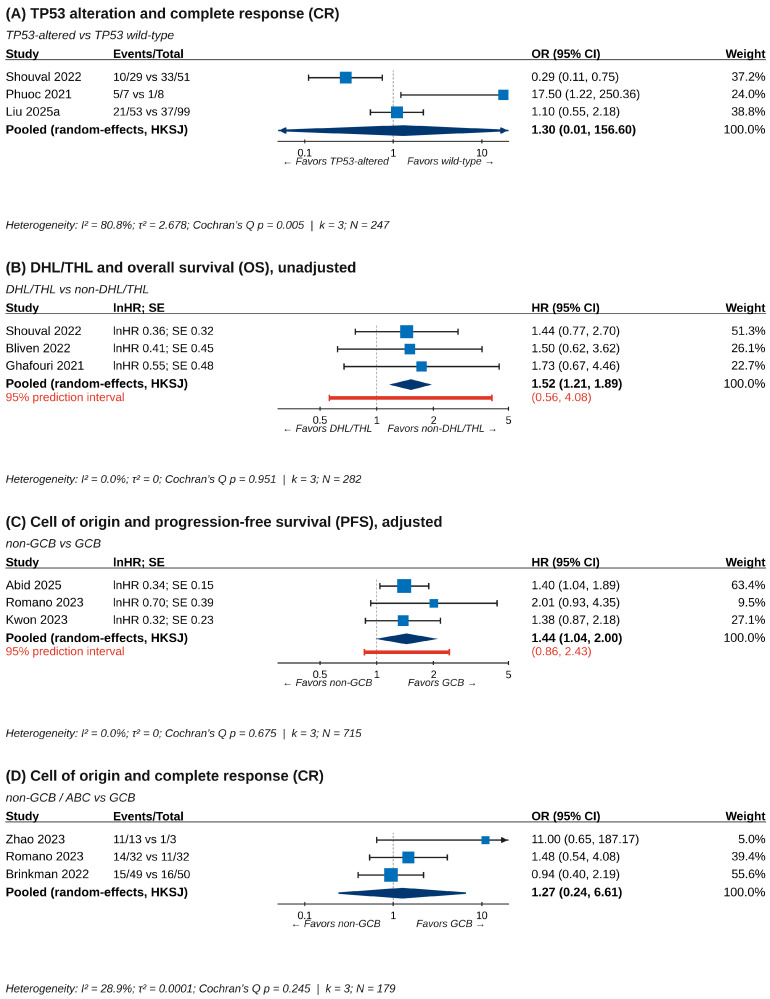
Forest plots of the four pooled meta-analyses of tumor genomic biomarkers and CD19 CAR T-cell outcomes in aggressive large B-cell lymphoma (aLBCL). Hazard ratio (HR) > 1 indicates worse outcomes, and odds ratio (OR) > 1 indicates higher response rates. (**A**) TP53 alteration and complete response (CR): three studies (Shouval 2022 [25], Phuoc 2021 [26], Liu 2025a [27]) contributing 247 CR-evaluable patients. Random-effects pooled odds ratio (OR) 1.30 (95% confidence interval, CI 0.01–156.60), with substantial between-study heterogeneity (I^2^ = 80.8%, τ^2^ = 2.68, Cochran’s Q *p* = 0.006); the pool is statistically inconclusive and dominated by Phuoc 2021 [26] (n = 15). (**B**) DHL/THL and overall survival (OS), unadjusted: three full-publication studies (Shouval 2022 [25], Bliven 2022 [28], Ghafouri 2021 [29]) contributing 282 patients. Random-effects pooled hazard ratio (HR) 1.52 (95% CI 1.21–1.89), with no detectable between-study heterogeneity (I^2^ = 0%, τ^2^ = 0); 95% prediction interval (PI) 0.56–4.08. (**C**) cell of origin (COO) and progression-free survival (PFS), adjusted: three studies (Abid 2025 [30], Romano 2023 [31], Kwon 2023 [32]) contributing 715 patients with adjusted multivariable Cox PFS hazard ratios. Random-effects pooled HR 1.44 (95% CI 1.04–2.00), with no detectable heterogeneity (I^2^ = 0%); 95% PI 0.86–2.43. (**D**) COO and CR: three studies (Romano 2023 [31], Zhao 2023 [33], Brinkman 2022 [34]) contributing 179 CR-evaluable patients. Random-effects pooled OR 1.27 (95% CI 0.24–6.61), with low-to-moderate heterogeneity (I^2^ = 28.9%, τ^2^ < 0.001); the pool is statistically inconclusive. Red bars represent the prediction interval. Blue squares represent the individual study effect. The dark blue diamond represents the pooled effect.

TP53-stratified survival outcomes were summarized narratively because fewer than three comparable studies provided extractable HRs for any TP53 survival endpoint. Shouval 2022 [25] reported worse OS among TP53-altered patients in both unadjusted analysis (HR 2.19; 95% CI, 1.18–4.10) and adjusted analysis (HR 2.03; 95% CI, 1.02–4.03), with directionally adverse but non-significant PFS estimates (unadjusted HR 1.40; 95% CI, 0.80–2.42; adjusted HR 1.51; 95% CI, 0.81–2.81) [Table 1 and Figure 2]. Gao 2023 [35] reported an adjusted OS HR of 1.90, but no 95% CI was available, precluding quantitative synthesis [Table 1 and Figure 2]. Sworder 2023 [39] reported an event-free survival (EFS) HR of 1.70 (95% CI, 1.00–2.70), but EFS was not pooled with PFS because of endpoint non-comparability [Table 1 and Figure 2]. Other TP53 reports lacked extractable HRs, had non-poolable endpoints, or provided insufficient biomarker-stratified outcome data (Supplementary Table S3.3-A; Supplementary Material S4). Overall, TP53-stratified survival data suggested a possible adverse association with OS after CD19 CAR T-cell therapy, but the evidence was too sparse and heterogeneous for pooled survival estimation.

### 3.5. MYC/BCL2/BCL6-Rearranged Double-Hit or Triple-Hit Lymphoma (DHL/THL)

DHL/THL status was represented in 12 biomarker-specific reports in the qualitative synthesis (Shouval 2022 [25], Karmali 2025 [9], Wang 2026 [2], Bliven 2022 [28], Phina-Zieben 2025 [42], Dodero 2025 [43], Locke 2024 [16], Olson 2023 [44], Shi 2023 [37], Chong 2020 [45], Ghafouri 2021 [29], Kwon 2023 [32]). Biomarker definitions and assay methods varied across studies; several reports used FISH or cytogenetic definitions of MYC plus BCL2 and/or BCL6 rearrangements, whereas others used composite high-grade B-cell lymphoma categories or gene-expression-based definitions and were retained for qualitative evidence mapping (Supplementary Table S3.2-B). Among the prespecified DHL/THL analyses, only the unadjusted OS analysis met the threshold of at least three comparable, non-overlapping studies for quantitative synthesis. Extracted DHL/THL outcome data are provided in Supplementary Table S3.3-B, and the feasibility rationale for each prespecified DHL/THL analysis is summarized in Supplementary Material S4.

#### 3.5.1. Unadjusted Overall Survival

Three full-text studies contributed extractable unadjusted OS hazard ratios for DHL/THL-positive vs. non-DHL/THL disease: Shouval 2022 [25], Bliven 2022 [28], and Ghafouri 2021 [29]. The pooled estimate indicated worse OS for DHL/THL-positive disease: HR 1.52 (95% CI, 1.21–1.89), with no detectable between-study heterogeneity (I^2^ = 0%; τ^2^ = 0; Q = 0.1, *p* = 0.951). The 95% PI was 0.56–4.08 (Table 1; Supplementary Material S5). Because the pooled HRs were unadjusted, this association may be affected by residual confounding and should be interpreted as exploratory.

Leave-one-out analyses preserved the adverse direction of effect, with two-study pooled HRs ranging from 1.46 to 1.60. Omission of Shouval 2022 [25] yielded HR 1.60 (95% CI, 0.65–3.96), omission of Bliven 2022 [28] yielded HR 1.52 (95% CI, 0.52–4.46), and omission of Ghafouri 2021 [29] yielded HR 1.46 (95% CI, 1.14–1.87) (Supplementary Table S6.2). The Ghafouri-omitted row also represents the cohort-exclusion sensitivity for the Ghafouri/UCLA overlap flag. Thus, the omission analyses did not reverse the adverse direction of the point estimate, but these k = 2 results were used only as descriptive influence diagnostics and not as formal evidence of robustness.

#### 3.5.2. Other DHL/THL Outcomes

Other DHL/THL-stratified outcomes were not pooled. Unadjusted PFS was not pooled because fewer than three comparable studies reported extractable PFS HRs, and EFS was not combined with PFS because of endpoint non-comparability. Adjusted PFS was not pooled because only two studies reported extractable adjusted PFS HRs. Adjusted OS was not pooled because only two studies reported extractable adjusted OS HRs. Complete-response analysis did not meet the pooling threshold because fewer than three studies provided clean, comparable 2 × 2 complete-response data by DHL/THL status. Biomarker-stratified safety data were sparse and were not quantitatively synthesized. Extracted study-level data for these non-pooled DHL/THL outcomes are provided in Supplementary Table S3.3-B, and the rationale for each non-pooled analysis is summarized in Supplementary Material S4.

### 3.6. Cell of Origin

COO was represented in 10 biomarker-specific reports in the qualitative synthesis (Abid 2025 [30], Shouval 2022 [25], Zhao 2023 [33], Brinkman 2022 [34], Ghafouri 2021 [29], Kwon 2023 [32], Locke 2024 [16], Manzar 2025 [46], Olson 2023 [44], Romano 2023 [31]). COO classification methods varied across studies and included immunohistochemistry-based algorithms, pathology-reported GCB vs. non-GCB classification, and gene-expression-based approaches (Supplementary Table S3.2-C). For quantitative synthesis, non-GCB and ABC-like COO were analyzed as the biomarker-positive group and compared with GCB COO. Among the prespecified COO analyses, adjusted PFS and complete response met the threshold of at least three comparable, non-overlapping studies. Other COO outcomes were not pooled because fewer than three comparable studies provided extractable effect estimates or complete-response data (Supplementary Material S4).

#### 3.6.1. Adjusted PFS

Three studies contributed extractable adjusted PFS hazard ratios for non-GCB/ABC vs. GCB COO: Abid 2025 [30], Romano 2023 [31], and Kwon 2023 [32]. Kwon 2023 [32] reported the HR in the opposite direction, as GCB vs. non-GCB; this estimate was inverted to align with the manuscript convention of non-GCB/ABC vs. GCB. The pooled estimate indicated worse adjusted PFS for non-GCB/ABC COO: HR 1.44 (95% CI, 1.04–2.00), with no detectable between-study heterogeneity (I^2^ = 0%; τ^2^ = 0; Q = 0.79, *p* = 0.675). The 95% PI was 0.86–2.43 (Figure 2C; Table 1; Supplementary Material S5).

Leave-one-out analyses preserved the direction of effect, with two-study pooled HRs ranging from 1.39 to 1.52. However, precision was inconsistent across omissions: exclusion of Romano 2023 [31] yielded HR 1.39 (95% CI, 1.28–1.52), whereas exclusion of Abid 2025 [30] yielded HR 1.52 (95% CI, 0.19–12.37) and exclusion of Kwon 2023 [32] yielded HR 1.47 (95% CI, 0.31–6.90). The Abid-omitted row also represents the cohort-exclusion sensitivity for the MD Anderson overlap/linkage flag. Accordingly, COO adjusted PFS analysis remained directionally consistent under omission analyses, but the k = 2 estimates were descriptive and sometimes highly imprecise (Supplementary Table S6.2).

#### 3.6.2. Complete Response

Three studies contributed extractable complete-response data by COO status: Zhao 2023 [33], Romano 2023 [31], and Brinkman 2022 [34]. The extracted complete-response data were 11/13 vs. 1/3 for Zhao 2023 [33], 14/32 vs. 11/32 for Romano 2023 [31], and 15/49 vs. 16/50 for Brinkman 2022 [34], comparing non-GCB/ABC with GCB COO. The pooled estimate was imprecise and statistically inconclusive: OR 1.27 (95% CI, 0.24–6.61), with low-to-moderate heterogeneity (I^2^ = 28.9%; τ^2^ = 0; Q = 2.81, *p* = 0.245) (Figure 2D; Table 1; Supplementary Material S5).

Study-level estimates were not consistent. Zhao 2023 [33] showed a large but highly imprecise estimate (OR 11.00; 95% CI, 0.65–187.18) driven by a 3-patient GCB comparator group, whereas Romano 2023 [31] showed a modest positive estimate (OR 1.49; 95% CI, 0.54–4.08) and Brinkman 2022 [34] showed a near-null estimate (OR 0.94; 95% CI, 0.40–2.19). Leave-one-out analysis showed that the COO complete-response estimate remained unstable and highly imprecise. Omission of Zhao 2023 [33] moved the point estimate toward 1.0, whereas omission of Romano 2023 [31] or Brinkman 2022 [34] moved it upward. These two-study estimates were interpreted descriptively only (Supplementary Material S6). Accordingly, the COO complete-response analysis was considered inconclusive.

#### 3.6.3. Other COO Outcomes

Other COO-stratified survival outcomes were not pooled. Unadjusted PFS was not pooled because only one study provided an extractable unadjusted PFS HR. Unadjusted OS and adjusted OS were not pooled because fewer than three comparable studies provided extractable HRs for either endpoint. Extracted study-level data for non-pooled COO outcomes are provided in Supplementary Table S3.3-C, and the rationale for each non-pooled analysis is summarized in Supplementary Material S4.

### 3.7. Heterogeneity and Sensitivity Analyses

Heterogeneity and model diagnostics for the four final pooled analyses are summarized in Table 1 and detailed in Supplementary Material S5. The DHL/THL unadjusted OS and COO adjusted PFS analyses showed no detectable between-study heterogeneity in the primary analyses (both I^2^ = 0%; τ^2^ = 0). In contrast, the TP53 complete response analysis showed substantial heterogeneity (I^2^ = 80.8%; τ^2^ = 2.68), reflecting markedly discordant study-level complete-response estimates, and the COO complete response analysis showed low-to-moderate inconsistency (I^2^ = 28.9%; τ^2^ = 0). PIs were reported for the DHL/THL unadjusted OS analysis (0.56–4.08) and the COO adjusted PFS analysis (0.86–2.43) and were not reported for the two complete-response pools because each binary-outcome analysis included only three studies with sparse event data. These statistical measures should be interpreted in the context of clinical and methodological heterogeneity in study setting, biomarker definitions, assay methods, endpoint definitions, and adjustment approaches.

Sensitivity analyses are reported in Supplementary Material S6. Because every primary pool included three studies, all leave-one-out and cohort-exclusion estimates were based on two-study models and were interpreted descriptively. Omission analyses preserved the adverse direction of the DHL/THL unadjusted OS and COO adjusted PFS point estimates, but several estimates were highly imprecise. The TP53 complete-response point estimate crossed 1.0 when Phuoc 2021 [26] was omitted, and the COO complete-response point estimate moved toward or away from 1.0 depending on the omitted study.

A low-risk-only sensitivity analysis was not feasible because no included study was rated as low overall risk of bias. Formal funnel-plot or regression-based publication-bias assessments were not performed because each pooled analysis included only three studies, below the prespecified threshold for such methods.

### 3.8. Cross-Biomarker Summary

Table 1 summarizes the four biomarker–endpoint combinations that met the prespecified threshold for quantitative synthesis. Two pooled survival analyses had 95% CIs excluding 1.0: DHL/THL-positive disease was associated with worse unadjusted OS (DHL/THL unadjusted OS: HR 1.52; 95% CI, 1.21–1.89), and non-GCB/ABC COO was associated with worse adjusted PFS (COO adjusted PFS: HR 1.44; 95% CI, 1.04–2.00). Both analyses had no detectable between-study heterogeneity, although their 95% PIs crossed 1.0, reflecting limited generalizability with only three contributing studies (Table 1; Figure 2B,C; Supplementary Material S5).

The two complete-response analyses were inconclusive. TP53 alteration and complete response (TP53 complete response) showed a highly unstable pooled estimate with substantial heterogeneity (OR 1.30; 95% CI, 0.01–156.60; I^2^ = 80.8%), driven chiefly by discordant study-level estimates including the small Phuoc 2021 [26] cohort. COO and complete response (COO complete response) also showed no consistent association (OR 1.27; 95% CI, 0.24–6.61), with sensitivity analyses demonstrating instability in both directions depending on which study was omitted (Table 1; Figure 2A,D; Supplementary Material S6).

Across all four analyses, the two-study omission models served only as descriptive in-fluence diagnostics and did not provide a reliable basis for inference (Supplementary Material S6). Accordingly, all pooled associations remain hypothesis-generating.

Prespecified subgroup analyses by CAR T-cell product, costimulatory domain, line of therapy, and study setting were not performed because each pooled analysis comprised only three studies, precluding meaningful within-stratum analysis. Meta-regression and formal publication-bias assessment were also not performed because each pooled analysis included only three studies. Per-study contribution tables for the four final pooled analyses are provided in Supplementary Material S7.

## 4. Discussion

### 4.1. Principal Findings and Interpretation

This systematic review and exploratory meta-analysis found that evidence for tumor genomic biomarkers after CD19-directed CAR T-cell therapy in aLBCL remains sparse, heterogeneous, and inconsistently reported. Although 26 studies were included in the qualitative synthesis, only four biomarker–endpoint combinations met the prespecified threshold for quantitative pooling. The limited number of poolable biomarker–endpoint combinations underscores the fragmented and inconsistent nature of biomarker-stratified outcome reporting in the CAR T-cell literature.

Among the four pooled analyses, the most informative signals were observed for survival outcomes. DHL/THL-positive disease was associated with worse unadjusted OS, and non-GCB/ABC COO was associated with worse adjusted PFS. These findings are directionally consistent with the established adverse prognostic relevance of DHL/THL status and COO classification in conventionally treated aggressive B-cell lymphoma [47,48]. However, both analyses included only three studies, and both 95% PIs crossed 1.0, limiting generalizability. These findings should therefore be interpreted as exploratory and hypothesis-generating rather than definitive.

The complete-response analyses were inconclusive. The pooled TP53–CR and COO–CR estimates were highly imprecise and did not support a consistent association with initial CR. For TP53, sensitivity analysis showed that the pooled estimate was unstable, with a small outlier cohort capable of shifting the direction of effect. These results do not establish absence of biological relevance. Rather, they indicate that the available data are insufficient to determine whether TP53 alteration or COO influences initial response after CAR T-cell therapy. These biomarkers may be more relevant to response durability, relapse risk, PFS, or OS than to early response, but the available biomarker-stratified survival data were too sparse to test this hypothesis formally.

Overall, the findings suggest that traditional LBCL biomarkers may retain prognostic relevance after CAR T-cell therapy, particularly for survival or disease-control endpoints. The current evidence is nonetheless insufficient to support use of TP53 alteration, DHL/THL status, or COO classification as standalone predictors of CAR T-cell outcome or as criteria for CAR T-cell selection. Unlike prior narrative reviews of CD19 CAR T-cell biomarkers, which have focused on broad summaries of single-study findings without formal quantitative pooling, the present review applies prespecified meta-analytic methods to identify which biomarker–outcome combinations currently support quantitative synthesis and which remain insufficiently reported for pooling. This complementary contribution highlights specific evidence gaps that prior narrative reviews could not quantify.

### 4.2. Comparison with Previous Studies

Pivotal CD19 CAR T-cell trials and large real-world series have established that CAR T-cell therapy induces durable remissions in a subset of patients with R/R LBCL, while also demonstrating substantial heterogeneity in response, relapse, survival, and toxicity [6,7,8,49,50,51]. Prior CAR T-cell reviews and biomarker-focused syntheses have largely addressed product-level efficacy, safety, real-world effectiveness, or clinical prognostic factors such as tumor burden, lactate dehydrogenase, inflammatory markers, bridging therapy, and CAR T-cell expansion, rather than systematically pooling tumor genomic biomarker strata [10,52,53]. Tumor genomic biomarkers have predominantly been reported in small subgroups, exploratory analyses, or incompletely stratified datasets.

The present review adds to this literature by systematically mapping biomarker-stratified outcomes and identifying which biomarker–endpoint combinations are currently quantifiable. In conventional chemoimmunotherapy-treated LBCL, TP53 alteration, DHL/THL status, and non-GCB/ABC COO have been associated with adverse outcomes [13,47,48,54,55]. Our findings suggest that these biomarkers may not translate uniformly into the CAR T-cell setting. DHL/THL and COO showed exploratory survival signals, whereas TP53 and COO were not consistently associated with initial CR. This pattern is compatible with the possibility that some tumor-intrinsic biomarkers influence post-CAR T-cell disease control or relapse risk more than early response.

Compared with previous product-level CAR T-cell reviews, the present review therefore highlights a distinct evidence gap: the absence of standardized biomarker-stratified reporting [10,53]. Overall, CAR T-cell efficacy data are not sufficient to define reliable genomic biomarker subgroups. The principal contribution of this review is to quantify rather than narratively assert how little of the current biomarker–outcome literature is poolable and to map which biomarker–endpoint combinations are presently quantifiable and those that remain limited to narrative evidence.

### 4.3. Clinical Implications

These findings should not change current clinical practice. CD19-directed CAR T-cell therapy should remain a treatment option for eligible patients with R/R aLBCL, including those with high-risk tumor biology [6,7,8,48]. The available evidence does not justify using TP53 alteration, DHL/THL status, or COO classification as standalone criteria to exclude patients from CAR T-cell therapy, select a specific product, or alter treatment strategy.

These biomarkers may nonetheless be useful for risk communication, trial stratification, and hypothesis generation. DHL/THL status and non-GCB/ABC COO may identify patients at higher risk of inferior survival or disease-control outcomes after CAR T-cell therapy, but these exploratory signals require confirmation in larger datasets with standardized biomarker definitions and adjusted survival modeling. Because the TP53 and COO complete-response pools were uninformative rather than null, a biomarker that has not been shown to predict initial response cannot be assumed to be irrelevant: it may still influence durability of response, relapse risk, or post-CAR T-cell survival, and should not be dismissed on the basis of complete-response data alone.

### 4.4. Strengths and Limitations

This review has several strengths. It followed a prospectively registered protocol and was conducted and reported in accordance with the PRISMA 2020 statement [19]. It focused on tumor genomic biomarkers with established biological relevance in LBCL-TP53 alteration, DHL/THL status, COO classification, and complex karyotype. It applied conservative pooling criteria, requiring at least three comparable, non-overlapping studies for each biomarker–endpoint combination; adjusted and unadjusted hazard ratios were not pooled together, EFS was not combined with PFS, and CR was not combined with ORR. These decisions reduced the number of pooled analyses but preserved their clinical and methodological interpretability. The k ≥ 3 threshold was a pragmatic minimum that avoided two-study primary meta-analyses, but even the resulting three-study pools remained exploratory.

The principal limitation is the small number of poolable studies. Each final meta-analysis included only three studies, which limited precision, precluded subgroup analysis, meta-regression, and formal publication-bias assessment, and constrained interpretation of heterogeneity statistics, PIs, and sensitivity analyses. In addition, PIs could be reported only for the two hazard-ratio pools, not for the two complete-response pools, because the binary-outcome analysis each included only three studies with sparse event data, further limiting the assessment of generalizability for these endpoints. Although the DHL/THL unadjusted OS and COO adjusted PFS analysis retained directionally adverse point estimates after individual study omission, several two-study sensitivity estimates were highly imprecise; the TP53 complete response and COO complete response analyses remained statistically uninformative because sensitivity estimates were unstable or extremely imprecise. Moreover, the evidence base varied in study setting, publication format, biomarker definitions and assay methods, endpoint definitions, and adjustment approaches; and many studies lacked biomarker-specific hazard ratios, 95% CIs, adjustment covariates, or complete 2 × 2 response data. Risk of bias was moderate or high for every included study, with none classified as low risk on the QUIPS tool [20]. Because the DHL/THL OS pool relied on unadjusted HRs, its exploratory association may be affected by residual confounding Finally, this review addressed tumor genomic biomarkers only; other determinants of CAR T-cell outcomes, including tumor burden, lactate dehydrogenase, inflammatory markers, bridging therapy, lymphodepletion, CAR T-cell expansion, T-cell fitness, antigen expression, and the immune microenvironment, were not consistently extractable and could not be incorporated into pooled multivariable models [10,52,53].

### 4.5. Future Directions

Future CAR T-cell trials and registries should incorporate predefined biomarker analysis plans and harmonized reporting standards [56,57]. Studies should report biomarker-evaluable denominators, assay methods, biomarker definitions, CR and ORR by biomarker status, PFS and OS hazard ratios with 95% CIs, adjustment covariates, CAR T-cell product, line of therapy, follow-up duration, and any cohort overlap with prior reports. For TP53, future studies should distinguish mutation, deletion, biallelic inactivation, and variant allele frequency where feasible. For DHL/THL, studies should separately characterize MYC/BCL2, MYC/BCL6, and triple-hit biology and avoid merging rearrangement-defined DHL/THL with broader HGBCL categories unless explicitly justified. For COO, studies should report whether classification was assigned by immunohistochemistry, pathology report, or gene-expression profiling, the last of which may be more biologically informative [48,58]. Complex karyotype, which no eligible study reported with extractable CAR T-cell outcome data, should be reported with defined cytogenetic criteria so that its prognostic role can eventually be assessed.

Future analyses should also distinguish biomarkers associated with initial response from those associated with response durability, relapse, PFS, and OS, since CAR T-cell therapy can induce early responses even in high-risk disease while longer-term relapse risk may remain more strongly governed by tumor biology. Larger registry-based analyses, harmonized multicenter cohorts, and individual patient-data meta-analyses may ultimately be required to determine whether tumor genomic biomarkers, alone or integrated with clinical, immunologic, and treatment-related variables, can meaningfully refine CAR T-cell risk stratification [59,60].

## 5. Conclusions

This systematic review with exploratory meta-analysis shows that tumor genomic biomarker evidence after CD19-directed CAR T-cell therapy in aLBCL remains fragmented and insufficiently standardized. Exploratory pooled analyses suggest that DHL/THL status may be associated with worse unadjusted OS and that non-GCB/ABC COO may be associated with inferior adjusted PFS, although the 95% PI crossed 1.0 for both analyses. The pooled TP53 and COO complete-response analyses were inconclusive, and no eligible study provided extractable complex-karyotype–stratified CAR T-cell outcome data.

These findings should be interpreted with caution and not used as definitive evidence for biomarker-guided CAR T-cell selection. TP53 alteration, DHL/THL status, and COO remain biologically important, hypothesis-generating markers rather than validated standalone predictors of CAR T-cell outcome. Standardized, prospective, biomarker-stratified reporting of CAR T-cell outcomes is needed to determine whether tumor genomic features can guide CAR T-cell risk stratification, trial design, or post-infusion management.

## Figures and Tables

**Table 1:** Cross-biomarker summary of final pooled meta-analyses.

Biomarker/Endpoint	Studies Included (k; n)	Pooled Estimate (95% CI)	Heterogeneity	95% PI
TP53-mutant/deleted vs. wild-type; CR (OR)	Shouval 2022 [25]; Phuoc 2021 [26]; Liu 2025a [27] (k = 3; n = 247 CR-evaluable)	OR 1.30 (0.01–156.60)	I^2^ = 80.8%; τ^2^ = 2.68; Q = 10.4 (*p* = 0.006)	Not shown
DHL/THL-positive vs. non-DHL/THL; unadjusted OS (HR)	Shouval 2022 [25]; Bliven 2022 [28]; Ghafouri 2021 [29] (k = 3; n = 282)	HR 1.52 (1.21–1.89)	I^2^ = 0%; τ^2^ = 0; Q = 0.1 (*p* = 0.951)	0.56–4.08
Non-GCB/ABC vs. GCB; adjusted PFS (HR)	Abid 2025 [30]; Romano 2023 [31]; Kwon 2023 [32] (k = 3; n = 715)	HR 1.44 (1.04–2.00)	I^2^ = 0%; τ^2^ = 0; Q = 0.79 (*p* = 0.675)	0.86–2.43
Non-GCB/ABC vs. GCB; CR (OR)	Zhao 2023 [33]; Romano 2023 [31]; Brinkman 2022 [34] (k = 3; n = 179 CR-evaluable)	OR 1.27 (0.24–6.61)	I^2^ = 28.9%; τ^2^ = 0; Q = 2.81 (*p* = 0.245)	Not shown

Notes: The four pools shown were the only biomarker–endpoint combinations that met the prespecified threshold of at least three non-overlapping studies for primary quantitative synthesis. HR > 1 indicates worse survival in the biomarker-positive or non-GCB/ABC group. For CR analyses, OR > 1 indicates higher odds of CR in the biomarker-positive or non-GCB/ABC group. The null value is 1.0 for both HRs and ORs. Pooled estimates used random-effects models with REML τ^2^ estimation and HKSJ confidence-interval correction. Prediction intervals (PI) are shown for HR-based pools only; they were not shown for CR pools because each binary-outcome pool included k = 3 studies with sparse event data. Kwon 2023 [32] was inverted from the reported GCB vs. non-GCB direction to match the non-GCB/ABC vs. GCB convention. Liu 2025a [27] refers to the TP53 CR abstract record; detailed identifiers and per-study contribution data are provided in Supplementary Material S7.

**Table 2:** Summary of the included evidence-based and study characteristics.

Characteristic	Summary
Included evidence-based	26 unique studies were included in the qualitative synthesis. Full per-study characteristics are provided in Supplementary Table S3.1.
Publication format	17 full-text articles/research letters and 9 conference abstracts.
Geographic coverage	Cohorts were reported from North America, Europe, and Asia, including multinational datasets.
Study design and setting	Predominantly retrospective real-world cohorts, registries, or EHR-derived analyses; a smaller number of trial-derived or exploratory biomarker analyses were included.
CAR T-cell products	Commercial CD19 CAR T-cell products were represented, most commonly axi-cel, tisa-cel, and liso-cel; several studies used mixed/not reported product cohorts and China-based cohorts using relma-cel or other CD19 CAR T-cell products were also present.
Line of therapy	Most studies evaluated third-line or later R/R aLBCL; a smaller number addressed second-line or mixed-line treatment settings.
Biomarker categories	TP53 alteration, MYC/BCL2/BCL6 rearrangement-defined DHL/THL, and COO were represented. Biomarker categories were not mutually exclusive. No eligible study provided extractable complex-karyotype-stratified CD19 CAR T-cell outcome data.
Biomarker-specific data availability	Final analytic datasets contained TP53 data in 13 rows across 11 biomarker-specific reports, DHL/THL data in 16 rows across 13 biomarker-specific reports, and COO data in 11 rows across 10 biomarker-specific reports. These counts are biomarker-sheet counts and may include separate adjusted/unadjusted rows for the same study.
Outcome types	Reported outcomes included CR/ORR, PFS or event-free survival (EFS), OS, DOR, CRS/ICANS, and TRM. Definitions, adjustment approaches, and biomarker assay methods varied across studies.
Quantitatively poolable evidence	Only 4 biomarker–endpoint combinations were suitable for meta-analysis: TP53 alteration/CR, DHL/THL/unadjusted OS, COO/adjusted PFS, and COO/CR; each pooled analysis included three studies.

Note: This compact main-text table summarizes the qualitative-synthesis evidence base. Full per-study characteristics are provided in Supplementary Table S3.1.

## Data Availability

No new data were created in this study. All data supporting the reported results were extracted from previously published articles, which are cited in the References section. Extracted datasets and summary tables generated during this study are available from the corresponding author upon reasonable request.

## Supplementary Materials

The following supporting information can be downloaded at: https://www.mdpi.com/article/10.3390/genes17070752/s1, Supplementary Material S1: Complete search strategies; Supplementary Material S2: PRISMA Checklist and Screening Eligibility Support; Supplementary Material S3: Study characteristics, biomarker definitions, extracted outcomes, and QUIPS risk of bias; Supplementary Material S4: Qualitative synthesis feasibility table; Supplementary Material S5: Detailed model output for final pooled analyses; Supplementary Material S6: Sensitivity analyses for the final pooled meta-analyses; Supplementary Material S7: Per-study contribution tables for pooled analyses; Supplementary Material S8: CART LBCL Analysis Code.R. References [61,62,63,64,65,66,67,68,69,70] are cited in the supplementary materials.

## Abbreviations

The following abbreviations are used in this manuscript:

ABC	Activated B-cell-like
aBCL	Aggressive B-cell lymphoma
Is noaLBCL	Aggressive large B-cell lymphoma
ASCT	Autologous stem cell transplantation
ASTCT	American Society for Transplantation and Cellular Therapy
Axi-cel	Axicabtagene ciloleucel
CAR T	Chimeric antigen receptor T cell
COO	Cell of Origin
CR	Complete response
CRS	Cytokine release syndrome
ctDNA	Circulating tumor deoxyribonucleic acid
DHL	Double-hit lymphoma
DLBCL	Diffuse large B-cell lymphoma
DOR	Duration of response
ECOG	Eastern Cooperative Oncology Group
EFS	Event-free survival
EHR	Electronic health record
FISH	Fluorescence in situ hybridization
GCB	Germinal center B-cell-like
GEP	Gene expression profile
HGBCL	High-grade B-cell lymphoma
HKSJ	Hartung–Knapp–Sidik–Jonkman
HR	Hazard ratio
ICANS	Immune effector cell-associated syndrome
IHC	Immunohistochemistry
Liso-cel	Lisocabtagene maraleucel
NHL	Non-Hodgkin’s lymphoma
OR	Odds ratio
OS	Overall survival
PFS	Progression-free survival
PI	Prediction interval
PMBCL	Primary mediastinal B-cell lymphoma
PRISMA	Preferred Reporting Items for Systematic Reviews and Meta-Analyses
QUIPS	Quality In Prognosis Studies
R CHOP	Rituximab, cyclophosphamide, doxorubicin, vincristine, and prednisone
REML	Restricted maximum likelihood
R/R	Relapsed or refractory
THL	Triple-hit lymphoma
Tisa-cel	Tisagenlecleucel
TRM	Treatment-related mortality
vs	Versus
WT	Wild type
