# Supplementary material for: Tumor Genomic Biomarkers as Prognostic Modifiers of Outcomes Following CD19 CAR T-Cell Therapy in Aggressive Large B-Cell Lymphoma: A Systematic Review and Exploratory Meta-Analysis
Source: Genes (Basel). 2026 Jun 30;17(7):752. doi: 10.3390/genes17070752 (PMC13409552; doi:10.3390/genes17070752)
Supplement: Supplementary file 1 [file genes-17-00752-s001.zip › Supplementary Material S1. Complete search strategies.pdf]

Supplementary Material S1. Complete search strategies

This supplement reports the search sources, search blocks, Web of Science query architecture, targeted PubMed citation lookup, and record accounting used for the PRISMA flow. It distinguishes systematic database searches from targeted full-text retrieval during citation chasing.

S1.1 Search overview and record counts

Systematic database searches were conducted in April-May 2026 without language restrictions at the search-execution step. No language restrictions were applied at the search step or during screening. All 182 unique records identified through the systematic search were English-language records, and no studies were excluded on the basis of language at either the title-and-abstract or full-text screening stages.

Source	Platform/vendor	Search role	Date	Records
MEDLINE	ProQuest Dialog	Four-block systematic search: population, intervention, biomarker, and exclusion filter	7 April 2026	22
Embase	ProQuest Dialog	Four-block systematic search: population, intervention, biomarker, and exclusion filter	7 April 2026	67
Web of Science Core Collection with BIOSIS Previews	Clarivate	One main Topic-field search plus four focused biomarker searches; results deduplicated within platform	4 Apri 2026	116 unique records after within-platform deduplication of 259 query-level records
PubMed	NCBI/NLM	Targeted citation lookup during full-text retrieval	7 May 2026	2
Total entering cross-source deduplication	-	-	-	207

The PubMed records were retrieved through targeted citation chasing to confirm full-text articles or bibliographic details for cohorts identified during screening/full-text retrieval. PubMed was not searched with the four-concept-block strategy because the MEDLINE search through ProQuest Dialog covered MEDLINE-indexed PubMed content within the search date range.

S1.2 Strategy A: MEDLINE and Embase via ProQuest Dialog

Strategy A combined three inclusion blocks with AND and applied the exclusion block with NOT: (Population AND Intervention AND Biomarker) NOT Exclusions. Free-text terms were searched in title, abstract, identifier, subject, and other platform-appropriate fields; controlled vocabulary was applied where available using MeSH for MEDLINE and Emtree for Embase.

Block	Terms used
1. Population: aggressive LBCL	LBCL; Large B(-)cell Lymphoma(-s); DLBCL; Lymphoma w/4 Diffuse w/2 B(-)Cell; HGBCL; High(-)Grade B(-)cell Lymphoma; tFL; Transformed Follicular Lymphoma; PMBCL; Primary Mediastinal B(-)cell Lymphoma. Controlled vocabulary: Diffuse Large B cell Lymphoma; Double-Hit Lymphoma; Primary Mediastinal Large B cell Lymphoma; Lymphoma, Large B-Cell, Diffuse.
2. Intervention: CD19 CAR-T therapy	(CAR(-)T OR Chimeric Antigen Receptor T-cell) AND (CD19 OR CD-19 OR Anti(-)CD19 OR Anti(-)CD-19); axicabtagene ciloleucel; axi-cel; Yescarta; Yikaida; tisagenlecleucel; tisa-cel; Kymriah; lisocabtagene maraleucel; liso-cel; Breyanzi. Controlled vocabulary: Chimeric Antigen Receptor T-cell Immunotherapy; Axicabtagene Ciloleucel; Tisagenlecleucel; Lisocabtagene Maraleucel; Immunotherapy, Adoptive; Receptors, Chimeric Antigen.
3. Biomarkers: tumor genomic biomarkers	TP53; Tumor Protein p53; MYC; BCL2; BCL6; Double(-)Hit Lymphoma; DHL; Triple(-)Hit Lymphoma; THL; Overexpression; Over-Expression; Cell of Origin; COO; Germinal Center B-cell-like; GCB; Activated B-cell-like; ABC; Complex Karyotype; Genomic or Molecular w/1 Alteration*, Biomarker*, Deletion*, or Mutation*; Tumor/Tumour Genomics; Cytogenetic; Molecular Subtype; Rearrangement*; Re-Arrangement*.
4. Exclusions	Child(-ren), Pediatric(s), or Paediatric(s) in title/identifier/subject/abstract fields; preclinical, nonclinical, nonhuman, animal, mouse, mice, murine, rat, or rodent terms in title/identifier/subject fields; and case reports, case series, reviews, comments, correspondence, editorials, letters, notes, short surveys, or topics in title/identifier/subject/document-type fields.

Strategy A yielded 89 ProQuest Dialog records: MEDLINE n = 22 and Embase n = 67.

S1.3 Targeted PubMed citation lookup

PubMed was used for targeted citation lookup during full-text retrieval, not as a separate comprehensive database search. The two PubMed records entered the combined record set because they provided full-text/bibliographic confirmation for cohorts identified during screening or citation chasing.

Tracker ID	Record retrieved through PubMed lookup	Reason for inclusion in record accounting
PubMed-001	[29] Ghafouri et al. 2021; DOI: 10.1016/j.clml.2021.07.002	Full publication retrieved after the related PIRLS-084 conference abstract; used to confirm the University of California Los Angeles cohort report.
PubMed-002	[32] Kwon et al. 2023; DOI: 10.3324/haematol.2022.280805	Spanish GETH-TC/GELTAMO registry analysis retrieved during full-text/bibliographic verification.

These two records should not be interpreted as the yield of a parallel four-block PubMed search.

S1.4 Strategy B: Web of Science Core Collection with BIOSIS Previews

All Web of Science searches used Topic-field syntax (TS=), which searches title, abstract, author keywords, and Keywords Plus. Five queries were run: one main three-block query covering all biomarker classes and four focused supplemental queries for TP53, DHL/THL, cell of origin, and complex karyotype.

S1.4.1 Common Web of Science blocks

Population block: TS=("large B-cell lymphoma" OR "large B cell lymphoma" OR LBCL OR DLBCL OR "diffuse large B-cell lymphoma" OR HGBCL OR "high-grade B-cell lymphoma" OR PMBCL OR "primary mediastinal B-cell lymphoma" OR "transformed follicular lymphoma" OR tFL)
Main-query intervention block: TS=("CAR T" OR "CAR-T" OR "CAR T-cell*" OR "CAR-T-cell*" OR "chimeric antigen receptor T-cell*" OR "chimeric antigen receptor T cell*" OR "CD19 CAR" OR "anti-CD19 CAR" OR "axicabtagene ciloleucel" OR "axi-cel" OR "tisagenlecleucel" OR "tisa-cel" OR "lisocabtagene maraleucel" OR "liso-cel")
Supplemental-query intervention block: TS=("CAR T" OR "CAR-T" OR "CAR T-cell*" OR "CAR-T-cell*" OR "chimeric antigen receptor T-cell*" OR "chimeric antigen receptor T cell*" OR "axicabtagene ciloleucel" OR "axi-cel" OR "tisagenlecleucel" OR "tisa-cel" OR "lisocabtagene maraleucel" OR "liso-cel")

S1.4.2 Web of Science biomarker blocks and record counts

Query	Biomarker block	Intervention block used	Records
Main 3-block search	TS=(TP53 OR p53 OR "double hit" OR "triple hit" OR DHL OR THL OR (("MYC" NEAR/3 "BCL2") OR ("MYC" NEAR/3 "BCL6") OR ("BCL2" NEAR/3 "BCL6")) OR "cell of origin" OR COO OR GCB OR "germinal center B-cell-like" OR "germinal centre B-cell-like" OR ABC OR "activated B-cell-like" OR "activated B cell-like" OR "non-GCB" OR "non GCB" OR "complex karyotype")	Main-query intervention block	118
TP53-focused search	TS=(TP53 OR p53)	Supplemental-query intervention block	44
DHL/THL-focused search	TS=("double hit" OR "triple hit" OR DHL OR THL OR ("MYC" NEAR/3 "BCL2") OR ("MYC" NEAR/3 "BCL6") OR ("BCL2" NEAR/3 "BCL6"))	Supplemental-query intervention block	63
COO-focused search	TS=("cell of origin" OR COO OR GCB OR "germinal center B-cell-like" OR "germinal centre B-cell-like" OR ABC OR "activated B-cell-like" OR "activated B cell-like" OR "non-GCB" OR "non GCB")	Supplemental-query intervention block	33

Query	Biomarker block	Intervention block used	Records
Complex karyotype-focused search	TS=("complex karyotype")	Supplemental-query intervention block	1

The five Web of Science query-level outputs totaled 259 records (118 + 44 + 63 + 33 + 1). After within-platform deduplication, 116 unique Web of Science/BIOSIS records were exported for cross-source deduplication. NEAR/3 indicates that terms occur within three words of each other in either direction; * indicates right-hand truncation.
No Web of Science document-type exclusion is reported as a search limit. Non-eligible publication types were excluded during screening according to the prespecified eligibility criteria.

S1.5 Deduplication and PRISMA record accounting

The combined record total before cross-source deduplication was 207: MEDLINE n = 22, Embase n = 67, Web of Science/BIOSIS n = 116, and targeted PubMed citation lookup n = 2. After removing 25 duplicate records across the combined set, 182 unique records advanced to title-and-abstract screening.
The 25 duplicate-record count applies to the combined cross-source record set. It should not be described as a MEDLINE/Embase-only or ProQuest-only duplicate count.
