# Supplementary material for: Tumor Genomic Biomarkers as Prognostic Modifiers of Outcomes Following CD19 CAR T-Cell Therapy in Aggressive Large B-Cell Lymphoma: A Systematic Review and Exploratory Meta-Analysis
Source: Genes (Basel). 2026 Jun 30;17(7):752. doi: 10.3390/genes17070752 (PMC13409552; doi:10.3390/genes17070752)
Supplement: Supplementary file 1 [file genes-17-00752-s001.zip › Supplementary Material S2. PRISMA Checklist and Screening Eligibility Support (1).pdf]

Supplementary Material S2

PRISMA Checklist and Screening/Eligibility Support

Supplementary Table S2.1. PRISMA 2020 checklist

Item	Topic	Checklist item	Location in submission
1	Title	Identify the report as a systematic review.	Title page
2	Abstract	Present the abstract in accordance with PRISMA 2020 abstract guidance.	Structured abstract
3	Rationale	Describe the rationale for the review in the context of existing knowledge.	Introduction
4	Objectives	Provide an explicit statement of the review question/objectives, including population, intervention/exposure, comparator, and outcomes.	Introduction; Methods
5	Eligibility criteria	Specify inclusion and exclusion criteria and how studies were grouped for synthesis.	Methods; Supplementary Material S1
6	Information sources	Specify all databases, platforms, registers, websites, and other sources searched or consulted.	Methods; Supplementary Material S1
7	Search strategy	Present the complete search strategies for all databases, including filters and limits used.	Supplementary Material S1
8	Selection process	State how records and reports were screened, how many reviewers were involved, and how disagreements were resolved.	Methods; Supplementary Material S1
9	Data collection process	State how data were collected, how many reviewers collected data, and how disagreements were resolved.	Methods; Supplementary Material S1
10a–b	Data items	List and define all outcomes and other extracted variables; describe assumptions about missing or unclear information.	Methods; Supplementary Material S3
11	Risk of bias in studies	Specify the tool and process used to assess risk of bias for included studies.	Methods; Results; Supplementary Table S3.4
12	Effect measures	Specify effect measures used for each outcome.	Methods; Table 2
13a–f	Synthesis methods	Describe criteria for synthesis eligibility, preparation of data, tabulation/visualization methods, synthesis models, heterogeneity assessment, and sensitivity analyses.	Methods; Table 2; Supplementary Materials S4–S6
14	Reporting bias assessment	Describe methods used to assess risk of bias due to missing results, or state why formal assessment was not performed.	Methods; Results/Discussion
15	Certainty assessment	Describe methods used to assess certainty/confidence in the body of evidence, if performed.	Not formally performed; limitations discussed
16a–b	Study selection	Describe the results of the selection process, preferably using a flow diagram, and cite studies that were excluded with reasons.	Figure 1; Tables S2.2–S2.5
17	Study characteristics	Cite each included study and present its characteristics.	Table 1; Supplementary Table S3.1
18	Risk of bias in studies	Present risk-of-bias assessments for included studies.	Results; Supplementary Table S3.4
19	Results of individual studies	Present results of individual studies for each outcome.	Supplementary Table S3.3; Supplementary Material S7

Item	Topic	Checklist item	Location in submission
20a–d	Results of syntheses	Present results of each synthesis, including summary estimates, precision, heterogeneity, and sensitivity analyses.	Table 2; Figure 2; Supplementary Materials S5–S6
21	Reporting biases	Present assessments of risk of bias due to missing results, if assessed.	Not formally assessed because each meta-analysis included k = 3 studies
22	Certainty of evidence	Present certainty/confidence assessments for each outcome, if assessed.	Not formally assessed
23a–d	Discussion	Summarize findings, discuss limitations of evidence and review methods, interpret results, and discuss implications.	Discussion
24a–c	Registration and protocol	Provide registration information, protocol access, and amendments.	Methods; Supplementary Material S1
25	Support	Describe sources of financial or non-financial support and the role of funders/sponsors.	Funding/support statement
26	Competing interests	Declare competing interests of review authors.	Conflicts of interest statement
27	Availability of data, code, and materials	Report availability of data, extraction files, analysis code, and other materials.	Data availability statement; Supplementary Materials S3 and S8

Supplementary Table S2.2. Reports assessed for eligibility (n = 37)

Study/report [Reference #]	Publication format	Source	Eligibility disposition
Porpaczy 2021 [37]	Full-text article	Web of Science/BIOSIS	Included in qualitative synthesis
Dodero 2025 [43]	Full-text article	Web of Science/BIOSIS	Included in qualitative synthesis
Shouval 2022 25]	Full-text article	Web of Science/BIOSIS	Included in qualitative synthesis
Shouval 2021 [63]	Conference/meeting abstract	Web of Science/BIOSIS	Excluded after eligibility assessment
Sheng 2025 [47]	Conference/meeting abstract	Web of Science/BIOSIS	Included in qualitative synthesis
Tumuluru 2025 [62]	Full-text article	Web of Science/BIOSIS	Excluded after eligibility assessment
Shi 2023 [39]	Letter/research letter	Web of Science/BIOSIS	Included in qualitative synthesis
Xue 2024 [40]	Full-text article	Web of Science/BIOSIS	Included in qualitative synthesis
Locke 2024 [16]	Full-text article	Web of Science/BIOSIS	Included in qualitative synthesis
Bliven 2022 [28]	Full-text article	Web of Science/BIOSIS	Included in qualitative synthesis
Phina-Ziebin 2025 [42]	Full-text article	Web of Science/BIOSIS	Included in qualitative synthesis
Olson 2023 [44]	Full-text article	Web of Science/BIOSIS	Included in qualitative synthesis
Gao 2023 [35]	Full-text article	Web of Science/BIOSIS	Included in qualitative synthesis
Manzar 2025 [46]	Full-text article	Web of Science/BIOSIS	Included in qualitative synthesis
Liu 2025 [63]	Full-text article	Web of Science/BIOSIS	Excluded after eligibility assessment
Zhao 2023 [33]	Full-text article	Web of Science/BIOSIS	Included in qualitative synthesis
Wang 2026 [2]	Full-text article	Web of Science/BIOSIS	Included in qualitative synthesis
Karmali 2025 [9]	Full-text article	Web of Science/BIOSIS	Included in qualitative synthesis
Kuhnl 2024 [64]	Full-text article	Web of Science/BIOSIS	Excluded after eligibility assessment
Abid 2025 [30]	Conference/meeting abstract	Web of Science/BIOSIS	Included in qualitative synthesis
Liu 2025a [27]	Conference/meeting abstract	Web of Science/BIOSIS	Included in qualitative synthesis
Ding 2025 [65]	Conference/meeting abstract	Embase	Excluded after eligibility assessment
Liu 2025b [41]	Conference/meeting abstract	Embase	Included in qualitative synthesis
Iraola-Truchuelo 2023 [66]	Conference/meeting abstract	Embase	Excluded after eligibility assessment
Romano 2023 [31]	Conference/meeting abstract	Embase	Included in qualitative synthesis
Phina-Ziebin 2023 [42]	Conference/meeting abstract	Embase	Excluded after eligibility assessment
Frost 2023 [69]	Conference/meeting abstract	Embase	Excluded after eligibility assessment
Strati 2023 [70]	Conference/meeting abstract	Embase	Excluded after eligibility assessment
Sworder 2023 [36]	Full-text article	MEDLINE	Included in qualitative synthesis
Batlevi 2022 [38]	Conference/meeting abstract	Embase	Included in qualitative synthesis
Brinkman 2022 [34]	Conference/meeting abstract	Embase	Included in qualitative synthesis
Hill 2021 [69]	Conference/meeting abstract	Embase	Excluded after eligibility assessment
Jain 2021 [70]	Conference/meeting abstract	Embase	Excluded after eligibility assessment

Study/report [Reference #]	Publication format	Source	Eligibility disposition
Phuoc 2021 [26]	Conference/meeting abstract	Embase	Included in qualitative synthesis
Chong 2020 [45]	Conference/meeting abstract	Embase	Included in qualitative synthesis
Ghafouri 2021 [29]	Full-text article	Targeted PubMed lookup	Included in qualitative synthesis
Kwon 2023 [32]	Full-text article	Targeted PubMed lookup	Included in qualitative synthesis

Supplementary Table S2.3. Excluded reports with primary reasons (n = 11)

Study/report	Primary exclusion reason	Specific reason
Shouval 2021 [61]	Endpoint mismatch or superseded by more complete report	Superseded by Shouval 2022 full publication reporting the same MSKCC cohort.
Tumuluru 2025 [62]	Biomarker–outcome stratification unavailable	Immune-environment analysis did not provide extractable outcomes for prespecified biomarker contrasts.
Liu 2025 [63]	CAR T-cell product unspecified or not protocol eligible	Prognostic transcriptomic-signature report did not provide an eligible approved CD19 CAR T-cell-treated biomarker-outcome cohort.
Kuhnl 2024 [64]	Biomarker–outcome stratification unavailable	Radiotherapy-bridging cohort; protocol biomarker variables were not reported with extractable biomarker-stratified CAR T-cell outcomes.
Ding 2025 [65]	No protocol-specified tumor genomic biomarker analyzed	ZUMA-7 multi-omics tumor-microenvironment subtypes were outside the prespecified TP53, DHL/THL, COO, and complex-karyotype biomarker set.
Iraola-Truchuelo 2023 [66]	CAR T-cell product unspecified or not protocol eligible	Mixed bispecific-antibody/CAR T-cell resistance analysis without an eligible product-specific approved CD19 CAR T-cell biomarker-outcome dataset.
Phina-Ziebin 2023 [42]	Endpoint mismatch or superseded by more complete report	Superseded by the Phina-Ziebin 2025 DESCAR-T LYSA full publication.
Frost 2023 [67]	No protocol-specified tumor genomic biomarker analyzed	Clinical laboratory mutation panel analysis did not provide an eligible prespecified biomarker contrast for this review.
Strati 2023 [68]	No protocol-specified tumor genomic biomarker analyzed	Germline genetic/polygenic risk-score analysis was outside the prespecified tumor genomic biomarker set.
Hill 2021 [69]	Biomarker–outcome stratification unavailable	Molecular-feature analysis did not provide extractable outcomes for prespecified biomarker contrasts.
Jain 2021 [70]	No protocol-specified tumor genomic biomarker analyzed	Whole-genome resistance analysis focused on non-prespecified genomic drivers; no eligible prespecified biomarker contrast was available.

Note: One primary reason was assigned for PRISMA reporting when a report had more than one limitation.

Abbreviations: CAR T-cell, chimeric antigen receptor T-cell therapy; COO, cell of origin; DHL/THL, double-hit/triple-hit lymphoma; LBCL, large B-cell lymphoma; PRISMA, Preferred Reporting Items for Systematic Reviews and Meta-Analyses.
