# Supplementary material for: Tumor Genomic Biomarkers as Prognostic Modifiers of Outcomes Following CD19 CAR T-Cell Therapy in Aggressive Large B-Cell Lymphoma: A Systematic Review and Exploratory Meta-Analysis
Source: Genes (Basel). 2026 Jun 30;17(7):752. doi: 10.3390/genes17070752 (PMC13409552; doi:10.3390/genes17070752)
Supplement: Supplementary file 1 [file genes-17-00752-s001.zip › Supplementary Material S3. Study characteristics, biomarker definitions, extracted outcomes, and QUIPS risk of bias.pdf]

## Supplementary Material S3
## Study characteristics, biomarker definitions, extracted outcomes, and QUIPS risk of bias

This supplement summarizes the study-level characteristics, biomarker definitions, extracted outcome data, and overall QUIPS risk-of-bias ratings for the qualitative-synthesis evidence base. Biomarker categories are not mutually exclusive.

Study references in Supplementary Table S3.1 correspond to the numbered reference list in the main manuscript.

### Supplementary Table S3.1. Study-level characteristics of included studies

| Study / report | Publication type | Geography | Study design / setting | CAR T-cell product(s) | Line | Biomarker category | Outcome types reported | Ref. |
|---|---|---|---|---|---|---|---|---|
| Chong 2020 | Conference abstract | Multinational | Retrospective cohort; real-world | Mixed / NR | NR | DHL/THL | ORR; PFS/OS (log-rank only) | [45] |
| Ghafouri 2021 | Full-text article | USA | Single-institution retrospective cohort; real-world | axi-cel | 3L+ | DHL/THL; COO | CR/ORR; PFS/OS; DOR; CRS/ICANS | [29] |
| Phuoc 2021 | Conference abstract | USA | Retrospective cohort; real-world | Mixed / NR | 3L+ | TP53 alteration | CR/ORR | [26] |
| Porpaczy 2021 | Full-text article | Austria | Single-institution retrospective cohort; real-world | Mixed / NR | 3L+ | TP53 alteration | OS | [37] |
| Batlevi 2022 | Conference abstract | USA | Retrospective cohort; real-world | Mixed / NR | 2L+ | TP53 alteration | Exploratory genomic–response only; no extractable endpoint | [38] |
| Bliven 2022 | Full-text article | USA | EHR-derived retrospective cohort (Flatiron); real-world | Mixed / NR | 3L+ | DHL/THL | OS | [28] |
| Brinkman 2022 | Conference abstract | USA | Retrospective cohort; real-world | Mixed / NR | 3L+ | COO | CR; PFS/OS (log-rank only) | [34] |
| Shouval 2022 | Full-text article | USA | Single-center retrospective cohort (MSKCC); real-world | axi-cel / tisa-cel / liso-cel | 3L+ | TP53 alteration; DHL/THL; COO | CR; PFS/OS | [25] |
| Gao 2023 | Full-text article | China | Retrospective cohort (Beijing Boren); real-world | Mixed China CD19 CAR T-cell products | Mixed | TP53 alteration | CR/ORR; PFS/OS | [35] |
| Kwon 2023 | Full-text article | Spain | Multicenter retrospective cohort (12 centers, GETH-TC/GELTAMO); real-world | axi-cel / tisa-cel | 3L+ | DHL/THL; COO | PFS; CRS/ICANS; TRM | [32] |
| Olson 2023 | Full-text article | Multinational | Translational analysis of phase 1 trial biopsies (TRANSCEND NHL 001); clinical trial | liso-cel | 3L+ | DHL/THL; COO | Response; PFS (narrative only) | [44] |
| Romano 2023 | Conference abstract | Multinational | Subgroup analysis of phase 3 RCT (ZUMA-7 2L subgroup); clinical trial | axi-cel | 2L | COO | CR/ORR; PFS/OS | [31] |
| Shi 2023 | Research letter (full-text) | China | Retrospective cohort (Beijing Boren); real-world | Mixed / NR | Mixed | TP53 alteration; DHL/THL | Response/CR (qualitative only) | [39] |
| Sworder 2023 | Full-text article | USA + Germany | Retrospective cohort (Stanford/MDACC/Essen, ctDNA STEP); real-world | axi-cel | 3L+ (≥2 prior lines) | TP53 alteration | EFS | [36] |
| Zhao 2023 | Full-text article | China | Prospective/retrospective single-center molecular cohort; real-world | axi-cel / relma-cel | Mixed (1L–4L prior) | COO | CR; CRS/ICANS | [33] |
| Locke 2024 | Full-text article | Multinational | Phase 3 RCT exploratory | axi-cel | 2L | DHL/THL; COO | EFS | [16] |

| Study / report | Publication type | Geography | Study design / setting | CAR T-cell product(s) | Line | Biomarker category | Outcome types reported | Ref. |
|---|---|---|---|---|---|---|---|---|
| | | | biomarker analysis (ZUMA-7); clinical trial | | | | | |
| Xue 2024 | Full-text article | China | Retrospective cohort; real-world | axi-cel / relma-cel | Mixed | TP53 alteration | Prognosis/survival (abstract preview only) | [40] |
| Abid 2025 | Conference abstract | USA | Single-center retrospective cohort (MD Anderson); real-world | Mixed / NR | 3L+ | COO | PFS/OS | [30] |
| Dodero 2025 | Full-text article | Italy | Prospective observational registry (CART SIE); real-world | axi-cel / tisa-cel | 3L+ | DHL/THL | OS; PFS/DOR; CRS/ICANS (qualitative) | [43] |
| Karmali 2025 | Full-text article | USA | Multicenter retrospective cohort (13 academic centers, ABC Consortium); real-world | axi-cel / tisa-cel / liso-cel | Mixed / 3L+ | DHL/THL | CR/ORR; PFS/OS; CRS/ICANS | [9] |
| Liu 2025a | Conference abstract | China | Retrospective cohort; real-world | Mixed / NR | NR | TP53 alteration | CR/ORR; PFS/OS | [27] |
| Liu 2025b | Conference abstract | China | Retrospective cohort; real-world | Mixed / NR | 3L+ | TP53 alteration | OS | [41] |
| Manzar 2025 | Full-text article | USA | Single-center retrospective cohort; real-world | axi-cel / liso-cel / tisa-cel | 3L+ / Mixed | COO | PFS / DSS / OS | [46] |
| Phina-Ziebin 2025 | Full-text article | France | Registry-based retrospective cohort (DESCAR-T LYSA); real-world | axi-cel / tisa-cel | 3L+ | DHL/THL | PFS/OS | [42] |
| Sheng 2025 | Conference abstract | China | Retrospective cohort; real-world | NR | NR | TP53 alteration | NR (no extractable data) | [47] |
| Wang 2026 | Full-text article | USA | Multicenter retrospective cohort (ABC Consortium); real-world | axi-cel / liso-cel | Mixed (2L / 3L / 4L+) | DHL/THL | PFS/OS | [2] |

Note: Complex karyotype was prespecified as an exploratory biomarker, but no eligible study provided extractable complex-karyotype-stratified CD19 CAR T-cell outcome data.

## Supplementary Table S3.2. Biomarker definitions and assay methods

## Supplementary Table S3.2-A. TP53 alteration

| Study / report | Assay method | Operational definition / comparison | Denominator / prevalence | Ref. |
|---|---|---|---|---|
| Batlevi 2022 | WES + RNA-Seq on tumor samples | Exploratory genomic complexity profiling; not a clean TP53-altered vs wild-type contrast | N = 54; pos/neg NR | [38] |
| Gao 2023 | Targeted sequencing (339-gene panel) | TP53 mutation in r/r DLBCL; CAR T-cell-treated survival subset | N = 65; pos = 32; neg = 33; prevalence 49.2% | [35] |
| Liu 2025a | NGS (TP53 mutation pre-screened as primary genomic variable of interest) | TP53 mutation vs wild-type | N = 152; pos = 53; neg = 99; prevalence 34.9% | [27] |
| Liu 2025b | NGS | TP53 mutation vs wild-type (10/22 patients) | N = 26; pos = 10; neg = 12 (22 of 26 with NGS data); prevalence 45.5% (of NGS-tested) | [41] |

| Study / report | Assay method | Operational definition / comparison | Denominator / prevalence | Ref. |
|---|---|---|---|---|
| Phuoc 2021 | NGS on pre-CAR T-cell tumor samples | TP53-mutant vs TP53 wild-type | N = 15; pos = 7; neg = 8; prevalence 46.7% | [26] |
| Porpaczy 2021 | Targeted NGS (161-gene panel; TP53 coding exons) | TP53 mutation in pre-CAR T-cell tumor; CAR T-cell cohort only | N = 29; pos = 10; neg = 19; prevalence 34.5% | [37] |
| Shi 2023 | Targeted deep sequencing (92 hematologic-related genes) | TP53 mutation among recurrent driver genes; association with CAR T-cell response explored | N = 84; pos = NR; neg = NR | [39] |
| Shouval 2022 | Targeted NGS (MSK-HemePACT) with copy-number assessment | TP53 alteration: mutation and/or copy-number alteration in pre-CAR T-cell tumor | N = 82 sequenced (153 total cohort); pos = 30; neg = 52; prevalence 36.6% (of sequenced) | [25] |
| Sworder 2023 | CAPP-seq deep sequencing (608 kb panel, 186 genes; Stanford STEP platform) | TP53 non-silent mutation (SNV/indel) by deep sequencing of plasma ctDNA and/or tumor tissue | N = 138; pos = NR; neg = NR | [36] |
| Xue 2024 | NGS for TP53 mutation and/or 17p deletion; DEL by IHC | TP53 alteration alone or co-occurring with double-expression lymphoma (MYC > 40%, BCL2 > 50%) | N = 73; pos/neg NR | [40] |

## Supplementary Table S3.2-B. DHL/THL

| Study / report | Assay method | Operational definition / comparison | Denominator / prevalence | Ref. |
|---|---|---|---|---|
| Bliven 2022 | FISH/cytogenetics from EHR (Flatiron) | DHL/THL: c-MYC plus BCL2 and/or BCL6 rearrangements | N = 76; pos/neg NR | [28] |
| Chong 2020 | FISH | DHL/THL by FISH (MYC + BCL2/BCL6 rearrangements) | N = 50; pos = 10; neg = 40; prevalence 20.0% | [45] |
| Dodero 2025 | Histology plus FISH where available | HGBL composite: HGBL-DH/TH, HGBL-NOS, and DLBCL with MYC/BCL6 rearrangement vs DLBCL | N = 432; pos = 78; neg = 354; prevalence 18.1% | [43] |
| Ghafouri 2021 | FISH (interphase dual-color break-apart probes for BCL-2, BCL-6, C-MYC, IgH) | DHL/THL: C-MYC rearrangement concurrent with BCL-2 and/or BCL-6 translocations (WHO 2017) | N = 53; pos = 14; neg = 39; prevalence 26.4% | [29] |
| Karmali 2025 | FISH/cytogenetics | DHL: MYC rearrangement plus BCL2 and/or BCL6 rearrangement; comparator non-DHL | N = 408; pos = 80; neg = 328; prevalence 19.6% | [9] |
| Kwon 2023 | Histopathology with FISH for HGBCL classification (WHO) | DH/TH HGBCL: high-grade B-cell lymphoma with MYC + BCL2 and/or BCL6 rearrangements (PMBCL excluded) | N = 307; pos = 45; neg = 262; prevalence 14.7% | [32] |
| Locke 2024 | Histology/cytogenetics plus NanoString biomarker analysis | HGBL composite (DH/TH disease) vs other disease; gene-expression high-grade feature included | N = 256 (axi-cel arm); pos = 46; neg = 210; prevalence 18.0% | [16] |
| Olson 2023 | RNA-seq double-hit gene-expression signature (DHITsig); histology table also reported | Double-hit gene-expression signature positive vs negative; not FISH-defined DHL/THL primary endpoint | N = 78; pos = 9; neg = NR; prevalence 11.5% | [44] |
| Phina-Ziebin 2025 | FISH/cytogenetics with national pathology review | HGBL (composite of HGBL-DH MYC-BCL2 + HGBL-TH + HGBL-DH MYC-BCL6 + HGBL-NOS, all FISH-confirmed) vs non-HGBL LBCL among CAR T-cell infused patients | N = 195 (DESCAR-T LYSA infused); pos = 60; neg = 135; prevalence 30.8% | [42] |
| Shi 2023 | FISH/cytogenetics; targeted sequencing cohort | DH/TH harboring MYC and BCL2/BCL6 translocations; whole cohort description | N = 84; pos = 13; neg = NR; prevalence not | [39] |

| Study / report | Assay method | Operational definition / comparison | Denominator / prevalence | Ref. |
|---|---|---|---|---|
| | | | reported | |
| Shouval 2022 | FISH/cytogenetics | Double-/triple-hit cytogenetic translocations vs non-DHL/THL | N = 138 evaluable for DHL/THL (153 total cohort); pos = 22; neg = 116; prevalence 15.9% | [25] |
| Wang 2026 | FISH/cytogenetics on initial diagnostic biopsy | DHL: MYC + BCL2 dual rearrangements; THL: MYC + BCL2 + BCL6 rearrangements | N = 466; pos = 80; neg = 386; prevalence 17.2% | [2] |

## Supplementary Table S3.2-C. Cell of origin

| Study / report | Assay method | Operational definition / comparison | Denominator / prevalence | Ref. |
|---|---|---|---|---|
| Abid 2025 | IHC Hans algorithm | Non-GCB vs GCB | N = 344; pos = 141; neg = 203; prevalence 41.0% | [30] |
| Brinkman 2022 | Pathology subtype (algorithm NR) | Non-GCB (combined ABC = 10 + NOS = 39) = 49 vs GCB = 50; NOS misclassification flag | N = 99; pos = 49; neg = 50; prevalence 49.5% | [34] |
| Ghafouri 2021 | IHC (algorithm not explicitly specified; likely Hans) | ABC/non-GCB vs GCB. Note: 12 NOS and 18 transformed DLBCL excluded from contrast | N = 41 (of 53 categorized); pos = 18; neg = 23; prevalence 43.9% | [29] |
| Kwon 2023 | Algorithm not explicitly stated (likely IHC); 44/307 unknown COO excluded | Non-GCB vs GCB | N = 263 (of 307; unknown excluded); pos = 90; neg = 173; prevalence 34.2% | [32] |
| Locke 2024 | Gene-expression COO classifier (NanoString) | Non-GCB-like / ABC-like vs GCB-like | N = 243 (of 256 axi-cel arm with COO); pos = 52; neg = 191; prevalence 21.4% | [16] |
| Manzar 2025 | Pathology subtype reporting (GCB vs non-GCB) | Non-GCB histology vs GCB | N = 46 (of 51 with COO); pos = 9; neg = 37; prevalence 17.6% | [46] |
| Olson 2023 | RNA-seq COO score/classifier | COO by gene-expression classifier; non-GCB/ABC vs GCB | N = 78; pos/neg NR | [44] |
| Romano 2023 | IHC Hans algorithm | Non-GCB vs GCB | N = 64 (ZUMA-7 2L subgroup); pos = 32; neg = 32; prevalence 50.0% | [31] |
| Shouval 2022 | IHC Hans algorithm | Non-GCB vs GCB | N = 148 (of 153 with COO); pos = 72; neg = 76; prevalence 47.1% | [25] |
| Zhao 2023 | Pathology-reported COO | Non-GCB vs GCB | N = 16; pos = 13; neg = 3; prevalence 81.3% | [33] |

## Supplementary Table S3.3. Extracted outcome data by biomarker

## Supplementary Table S3.3-A. TP53 alteration

| Study / report | Synthesis role | HR type | PFS/EFS HR (95% CI) | OS HR (95% CI) | CR data | ORR data | Safety / TRM | Ref. |
|---|---|---|---|---|---|---|---|---|
| Porpaczy 2021 | Qualitative only | Not reported | — | — | — | — | — | [37] |

| Study / report | Synthesis role | HR type | PFS/EFS HR (95% CI) | OS HR (95% CI) | CR data | ORR data | Safety / TRM | Ref. |
|---|---|---|---|---|---|---|---|---|
| Shouval 2022 | Quantitative + qualitative | Unadjusted | HR 1.40 (0.80–2.42) | HR 2.19 (1.18–4.10) | 10/29 vs 33/51 | — | — | [25] |
| Shouval 2022 | Quantitative + qualitative | Adjusted | HR 1.51 (0.81–2.81) | HR 2.03 (1.02–4.03) | — | — | — | [25] |
| Gao 2023 | Quantitative (ORR) + qualitative | Adjusted | — | HR 1.90 (95% CI NR) | 7/32 vs NR | 18/32 vs 27/33 | — | [35] |
| Shi 2023 | Qualitative only | Not reported | — | — | — | — | — | [39] |
| Xue 2024 | Qualitative only | Not extractable | — | — | — | — | — | [40] |
| Phuoc 2021 | Quantitative + qualitative | Not reported | — | — | 5/7 vs 1/8 | 7/7 vs 6/8 | — | [26] |
| Liu 2025a | Quantitative + qualitative | Not reported | — | — | 21/53 vs 37/99 | 30/53 vs 47/99 | — | [27] |
| Liu 2025b | Qualitative only | Not reported | — | — | — | — | — | [41] |
| Sworder 2023 | Qualitative only | Unadjusted (EFS) | EFS HR 1.70 (1.00–2.70) | — | — | — | — | [36] |
| Batlevi 2022 | Qualitative only | Not reported | — | — | — | — | — | [38] |

## Supplementary Table S3.3-B. DHL/THL

| Study / report | Synthesis role | HR type | PFS/EFS HR (95% CI) | OS HR (95% CI) | CR data | ORR data | Safety / TRM | Ref. |
|---|---|---|---|---|---|---|---|---|
| Shouval 2022 | Quantitative + qualitative | Unadjusted | — | HR 1.44 (0.77–2.71) | — | — | — | [25] |
| Karmali 2025 | Quantitative + qualitative | Adjusted (multivariable Cox) | HR 0.80 (0.50–1.30) | — | 39/80 vs 157/328 | 55/80 vs 216/328 | CRS ≥ G3: 14/61 vs NR; ICANS ≥ G3: 15/49 vs NR | [9] |
| Wang 2026 | Quantitative + qualitative | Adjusted | HR 1.30 (0.90–1.90) | HR 1.40 (1.00–2.00) | — | — | — | [2] |
| Bliven 2022 | Quantitative + qualitative | Unadjusted | — | HR 1.50 (0.60–3.50) | — | — | — | [28] |
| Bliven 2022 | Quantitative + qualitative | Adjusted | — | HR 1.70 (0.60–4.80) | — | — | — | [28] |
| Phina-Ziebin 2025 | Qualitative only | Not reported | — | — | — | — | — | [42] |
| Dodero 2025 | Qualitative only | Weighted comparison; HR not reported | — | — | — | — | CRS ≥ G3: 10/78 vs 24/354; ICANS ≥ G3: 6/78 vs 22/354 | [43] |
| Locke 2024 | Qualitative only | Unadjusted (EFS) | EFS HR 0.69 (0.38–1.25) | — | — | — | — | [16] |

| Study / report | Synthesis role | HR type | PFS/EFS HR (95% CI) | OS HR (95% CI) | CR data | ORR data | Safety / TRM | Ref. |
|---|---|---|---|---|---|---|---|---|
| Olson 2023 | Qualitative only | Not reported | — | — | — | — | — | [44] |
| Shi 2023 | Qualitative only | Not reported | — | — | — | — | — | [39] |
| Chong 2020 | Quantitative (ORR) + qualitative | Not reported | — | — | — | 5/10 vs 22/40 | — | [45] |
| Ghafouri 2021 | Quantitative + qualitative | Unadjusted | HR 2.16 (1.00–4.65) | HR 1.73 (0.74–4.91) | 7/14 vs 27/39 | 9/14 vs 29/39 | CRS ≥ G3: 1/14 vs 2/39; ICANS ≥ G3: 5/14 vs 7/39 | [29] |
| Kwon 2023 | Qualitative only | Not reported | — | — | — | — | CRS ≥ G3: 19/261 vs NR; ICANS ≥ G3: 30/261 vs NR; TRM 13/261 vs NR | [32] |

## Supplementary Table S3.3-C. Cell of origin

| Study / report | Synthesis role | HR type | PFS/EFS HR (95% CI) | OS HR (95% CI) | CR data | ORR data | Safety / TRM | Ref. |
|---|---|---|---|---|---|---|---|---|
| Shouval 2022 | Qualitative only | Unadjusted | — | HR 1.00 (0.62–1.60) | — | — | — | [25] |
| Zhao 2023 | Quantitative + qualitative | Not reported | — | — | 11/13 vs 1/3 | — | CRS ≥ G3: 2/13 vs 0/3; ICANS ≥ G3: 1/13 vs 0/3 | [33] |
| Locke 2024 | Qualitative only | Not reported | — | — | — | — | — | [16] |
| Olson 2023 | Qualitative only | Not reported | — | — | — | — | — | [44] |
| Manzar 2025 | Qualitative only | Not reported | — | — | — | — | — | [30] |
| Abid 2025 | Quantitative + qualitative | Adjusted | HR 1.40 (1.04–1.89) | HR 1.46 (1.03–2.07) | — | — | — | [31] |
| Romano 2023 | Quantitative + qualitative | Adjusted | HR 2.01 (0.93–4.35) | HR 1.73 (0.71–4.22) | 14/32 vs 11/32 | 24/32 vs 22/32 | — | [31] |
| Romano 2023 | Quantitative + qualitative | Unadjusted | HR 1.18 (0.61–2.28) | HR 0.82 (0.39–1.73) | 14/32 vs 11/32 | 24/32 vs 22/32 | — | [31] |
| Brinkman 2022 | Qualitative (CR 2×2) | Not reported | — | — | 15/49 vs 16/50 | — | — | [34] |
| Ghafouri 2021 | Qualitative only | Not reported (logistic regression OR only) | — | — | — | — | — | [29] |
| Kwon 2023 | Quantitative + qualitative | Adjusted | HR 1.38 (0.87–2.17) | — | — | — | — | [32] |

Note: For CR and ORR, values are shown as biomarker-positive events/total versus biomarker-negative events/total. HRs follow the manuscript direction-of-effect convention.

## Supplementary Table S3.4. Overall QUIPS risk-of-bias ratings

| Study / report | Biomarker category | Publication type | Overall QUIPS rating | Rationale / comment | Ref. |
|---|---|---|---|---|---|
| Chong 2020 | DHL/THL | Conference abstract | High | Abstract-only or limited-reporting record | [45] |
| Ghafouri 2021 | DHL/THL; COO | Full-text article | Moderate | Cohort overlap with Ghafouri 2021 abstract precursor; handled by overlap rule | [29] |
| Phuoc 2021 | TP53 alteration | Conference abstract | High | Abstract-only; n = 15 with sparse 2×2 cells driving outlier influence on TP53-05 | [26] |
| Porpaczy 2021 | TP53 alteration | Full-text article | High | Small CAR T-cell subgroup (n = 29); incomplete biomarker–outcome reporting; no poolable endpoint | [37] |
| Batlevi 2022 | TP53 alteration | Conference abstract | Moderate | Abstract-only; exploratory genomic-response analysis; no poolable endpoint | [38] |
| Bliven 2022 | DHL/THL | Full-text article | High | EHR-derived (Flatiron); DHL/THL vs non-DHL/THL denominators within CAR T-cell subgroup not reported | [28] |
| Brinkman 2022 | COO | Conference abstract | High | Abstract-only; no Cox HR; KM + log-rank only | [34] |
| Shouval 2022 | TP53 alteration; DHL/THL; COO | Full-text article | Moderate | MSKCC cohort overlaps with Shouval 2021 abstract precursor (TP53) and partially with Tumuluru 2025 MSKCC validation cohort; handled by overlap rule | [25] |
| Gao 2023 | TP53 alteration | Full-text article | Moderate | Beijing Boren cohort overlaps with Shi 2023; handled by overlap rule; HR lacks CI | [35] |
| Kwon 2023 | DHL/THL; COO | Full-text article | Moderate | Multicenter Spanish registry; overall rating from extraction tracker | [32] |
| Olson 2023 | DHL/THL; COO | Full-text article | High | Translational analysis of phase 1 trial biopsies; gene-expression definition not protocol-primary; no extractable HR/CI | [44] |
| Romano 2023 | COO | Conference abstract | Moderate | Conference-abstract analysis of ZUMA-7 2L axi-cel subgroup; both adjusted and unadjusted HRs reported | [31] |
| Shi 2023 | TP53 alteration; DHL/THL | Research letter (full-text) | High | Beijing Boren cohort overlaps with Gao 2023; no extractable HR/CI or 2×2; handled by overlap rule | [39] |
| Sworder 2023 | TP53 alteration | Full-text article | Moderate | ctDNA STEP analysis; EFS endpoint (not PFS), so not poolable in primary PFS pool | [36] |
| Zhao 2023 | COO | Full-text article | High | Small mechanistic single-center cohort (n = 16); no time-to-event HRs; sparse 2×2 cells in CR pool | [33] |
| Locke 2024 | DHL/THL; COO | Full-text article | Moderate | ZUMA-7 phase 3 trial exploratory analysis; overall rating from extraction tracker; EFS endpoint mismatch | [16] |
| Xue 2024 | TP53 alteration | Full-text article | High | Full text not attached; abstract preview only; no extractable HR/CI or 2×2; no poolable endpoint | [40] |
| Abid 2025 | COO | Conference abstract | Moderate | MD Anderson cohort; flagged for potential overlap with published MDACC subset; handled by overlap-cohort sensitivity | [30] |
| Dodero 2025 | DHL/THL | Full-text article | Moderate | Composite HGBL definition not strictly rearrangement-defined; no extractable HR/CI by primary DHL/THL definition | [43] |
| Karmali 2025 | DHL/THL | Full-text article | Moderate | ABC Consortium 13-center cohort overlaps with Wang 2026; handled by overlap rule (not pooled together) | [9] |
| Liu 2025a | TP53 alteration | Conference abstract | High | Abstract-only; large cohort (n = 152) with extractable CR/ORR 2×2; no Cox HR/CI | [27] |
| Liu 2025b | TP53 alteration | Conference abstract | High | Abstract-only; small cohort (n = 26); potential cohort overlap with Liu 2025a (same first author, same year, China); no extractable HR/CI | [41] |
| Manzar 2025 | COO | Full-text article | High | Small single-center cohort (n = 51); no Cox HR/CI; strong qualitative directional finding only | [46] |
| Phina-Ziebin | DHL/THL | Full-text article | Moderate | DESCAR-T LYSA registry supersedes Phina-Ziebin 2023 abstract precursor; handled by overlap | [42] |

| Study / report | Biomarker category | Publication type | Overall QUIPS rating | Rationale / comment | Ref. |
|---|---|---|---|---|---|
| 2025 | | | | rule | |
| Sheng 2025 | TP53 alteration | Conference abstract | High | Abstract source text not accessible in the screening package; no extractable data of any kind; retained for traceability only | [47] |
| Wang 2026 | DHL/THL | Full-text article | Moderate | ABC Consortium cohort overlaps with Karmali 2025; handled by overlap rule (not pooled together) | [2] |

Note: Overall ratings are shown as low, moderate, or high risk of bias; no study was rated low risk.

## Abbreviations

ABC, activated B-cell-like; axi-cel, axicabtagene ciloleucel; CAR T-cell, chimeric antigen receptor T-cell therapy; CI, confidence interval; COO, cell of origin; CR, complete response; CRS, cytokine release syndrome; DHL/THL, double-hit/triple-hit lymphoma; DOR, duration of response; DSS, disease-specific survival; EFS, event-free survival; EHR, electronic health record; FISH, fluorescence in situ hybridization; GCB, germinal center B-cell-like; HR, hazard ratio; ICANS, immune effector cell-associated neurotoxicity syndrome; IHC, immunohistochemistry; LBCL, large B-cell lymphoma; liso-cel, lisocabtagene maraleucel; NGS, next-generation sequencing; NR, not reported; ORR, overall response rate; OS, overall survival; PFS, progression-free survival; QUIPS, Quality in Prognosis Studies; RNA-Seq, RNA sequencing; tisa-cel, tisagenlecleucel; TRM, treatment-related mortality; WES, whole-exome sequencing.
