# Supplementary material for: Tumor Genomic Biomarkers as Prognostic Modifiers of Outcomes Following CD19 CAR T-Cell Therapy in Aggressive Large B-Cell Lymphoma: A Systematic Review and Exploratory Meta-Analysis
Source: Genes (Basel). 2026 Jun 30;17(7):752. doi: 10.3390/genes17070752 (PMC13409552; doi:10.3390/genes17070752)
Supplement: Supplementary file 1 [file genes-17-00752-s001.zip › Supplementary Material S4. Quantitative synthesis feasibility table.pdf]

## Supplementary Material S4

## Quantitative-synthesis feasibility table

This supplement summarizes the feasibility assessment for each prespecified biomarker–endpoint combination. Primary quantitative synthesis required at least three non-overlapping studies with a comparable biomarker contrast, endpoint, effect measure, and adjustment stratum. Time-to-event analyses required a hazard ratio with a 95% confidence interval; binary-response analyses required extractable 2×2 data. Adjusted and unadjusted hazard ratios were not combined, and event-free survival (EFS) was not pooled with progression-free survival (PFS). Each prespecified analysis is identified by an internal analysis ID (biomarker prefix plus sequence number); these IDs are retained here as the organizing key of the feasibility map. Study references in Supplementary Table S4.1 correspond to the numbered reference list in the main manuscript.

### Supplementary Table S4.1. Feasibility map for prespecified pooled analyses

| Analysis ID | Biomarker / comparison | Endpoint and effect measure | Decision | Feasibility rationale |
|---|---|---|---|---|
| **TP53-01** | TP53-mutant/deleted vs TP53 wild-type | PFS, unadjusted HR | **Not pooled** | Only Shouval 2022 [25] provided a complete unadjusted PFS HR with 95% CI. Other records reported log-rank results only, an EFS endpoint rather than PFS, or no extractable HR with 95% CI. |
| **TP53-02** | TP53-mutant/deleted vs TP53 wild-type | PFS, adjusted HR | **Not pooled** | Only Shouval 2022 [25] provided a complete adjusted PFS HR with 95% CI. No second or third comparable adjusted PFS estimate with a 95% CI was available. |
| **TP53-03** | TP53-mutant/deleted vs TP53 wild-type | OS, unadjusted HR | **Not pooled** | Only Shouval 2022 [25] provided a complete unadjusted OS HR with 95% CI. Other studies lacked an extractable OS HR with 95% CI. |
| **TP53-04** | TP53-mutant/deleted vs TP53 wild-type | OS, adjusted HR | **Not pooled** | Only Shouval 2022 [25] provided a complete adjusted OS HR with 95% CI. Gao 2023 [35] reported an adjusted OS HR without a 95% CI; Liu 2025b [41] reported Kaplan–Meier OS data without a Cox HR; other records lacked comparable adjusted OS estimates. |
| **TP53-05** | TP53-mutant/deleted vs TP53 wild-type | CR, OR | **Pooled** | Three non-overlapping studies provided extractable CR 2×2 data: Shouval 2022 [25], Phuoc 2021 [26], and Liu 2025a [27]. Threshold of k ≥3 non-overlapping studies met. |
| **DHL-01** | DHL/THL-positive vs non-DHL/THL | PFS, unadjusted HR | **Not pooled** | Two studies reported a complete unadjusted time-to-event HR with 95% CI: Ghafouri 2021 [29] (PFS) and Locke 2024 [16]. The Locke 2024 [16] estimate was an EFS endpoint (ZUMA-7 axi-cel arm), which was not pooled with PFS per the prespecified endpoint rule, leaving only one poolable unadjusted PFS HR. Other potentially relevant records reported adjusted PFS, log-rank results, or no extractable HR with 95% CI. |
| **DHL-02** | DHL/THL-positive vs non-DHL/THL | PFS, adjusted HR | **Not pooled** | Only two studies reported a complete adjusted PFS HR with 95% CI (Karmali 2025 [9]; Wang 2026 [2]), so k remained below the pooling threshold. These two estimates also likely draw on overlapping ABC Consortium cohorts, which would have precluded their joint inclusion even if a third estimate had been available. |

| Analysis ID | Biomarker / comparison | Endpoint and effect measure | Decision | Feasibility rationale |
|---|---|---|---|---|
| **DHL-03** | DHL/THL-positive vs non-DHL/THL | OS, unadjusted HR | **Pooled** | Three non-overlapping studies provided complete unadjusted OS HRs with 95% CIs: Shouval 2022 [25] (MSKCC), Bliven 2022 [28] (Flatiron EHR), and Ghafouri 2021 [29] (UCLA). Threshold of k ≥3 non-overlapping studies met. |
| **DHL-04** | DHL/THL-positive vs non-DHL/THL | OS, adjusted HR | **Not pooled** | Only two studies provided a complete adjusted OS HR with 95% CI (Bliven 2022 [28]; Wang 2026 [2]), so k remained below the pooling threshold. Adjusted and unadjusted HRs were not combined. |
| **DHL-05** | DHL/THL-positive vs non-DHL/THL | CR, OR | **Not pooled** | Fewer than three studies provided clean, comparable CR 2×2 data. ORR-only data were not substituted for CR. |
| **COO-01** | Non-GCB/ABC vs GCB | PFS, unadjusted HR | **Not pooled** | Only Romano 2023 [31] provided a complete unadjusted PFS HR with 95% CI. Other PFS estimates were adjusted or were not extractable with a 95% CI. |
| **COO-02** | Non-GCB/ABC vs GCB | PFS, adjusted HR | **Pooled** | Three studies provided complete adjusted PFS HRs with 95% CIs: Abid 2025 [30], Romano 2023 [31], and Kwon 2023 [32]. The Kwon 2023 [32] estimate was inverted to the non-GCB/ABC vs GCB convention (Cochrane Handbook §6.3.2 [20]). Threshold of k ≥3 met. |
| **COO-03** | Non-GCB/ABC vs GCB | OS, unadjusted HR | **Not pooled** | Only Shouval 2022 [25] and Romano 2023 [31] provided a complete unadjusted OS HR with 95% CI; k remained below the pooling threshold. |
| **COO-04** | Non-GCB/ABC vs GCB | OS, adjusted HR | **Not pooled** | Only Abid 2025 [30] and Romano 2023 [31] provided a complete adjusted OS HR with 95% CI; k remained below the pooling threshold. |
| **COO-05** | Non-GCB/ABC vs GCB | CR, OR | **Pooled** | Three non-overlapping studies provided extractable CR 2×2 data: Zhao 2023 [33], Romano 2023 [31], and Brinkman 2022 ]34]. Threshold of k ≥3 non-overlapping studies met. |

**Note.** The four analyses meeting the feasibility criteria and contributing to the quantitative synthesis were TP53-05, DHL-03, COO-02, and COO-05. All other prespecified analyses failed the k ≥ 3 non-overlapping-study threshold or lacked comparable extractable data. Complex karyotype was prespecified as an exploratory biomarker, but no eligible study provided extractable complex-karyotype-stratified CD19 CAR T-cell outcome data, so no complex-karyotype analysis was attempted. Publication-bias testing (funnel-plot asymmetry and related tests), meta-regression, and formal subgroup analyses were not performed because no pooled analysis included a sufficient number of studies (each pooled analysis comprised exactly three studies, below the conventional minimum of ten studies for funnel-plot-based and meta-regression methods [24].

**Abbreviations.** ABC, activated B-cell-like; CAR T-cell, chimeric antigen receptor T-cell therapy; CI, confidence interval; COO, cell of origin; CR, complete response; DHL/THL, double-hit/triple-hit lymphoma; EFS, event-free survival; GCB, germinal-centre B-cell-like; HR, hazard ratio; MSKCC, Memorial Sloan Kettering Cancer Center; OR, odds ratio; ORR, overall response rate; OS, overall survival; PFS, progression-free survival; UCLA, University of California, Los Angeles.
