# Supplementary material for: Tumor Genomic Biomarkers as Prognostic Modifiers of Outcomes Following CD19 CAR T-Cell Therapy in Aggressive Large B-Cell Lymphoma: A Systematic Review and Exploratory Meta-Analysis
Source: Genes (Basel). 2026 Jun 30;17(7):752. doi: 10.3390/genes17070752 (PMC13409552; doi:10.3390/genes17070752)
Supplement: Supplementary file 1 [file genes-17-00752-s001.zip › Supplementary Material S5. Detailed model output for final pooled analyses.pdf]

Supplementary Material S5

Detailed model output for final pooled analyses

This supplement provides model diagnostics and study-level inputs for the four final pooled analyses. Effects are presented on the original HR or OR scale for readability. Models were fitted on the natural-log scale and back-transformed for presentation as HRs or ORs. Study references in Supplementary Tables S5.1 and S5.2 correspond to the numbered reference list in the main manuscript.

Supplementary Table S5.1. Model-level diagnostics presented on the original effect scale

Pool ID	Biomarker/endpoint	Studies (k; n)	Pooled estimate (95% CI)	τ^2	I^2 (%)	Q (df; p)	95% prediction interval
TP53-05	TP53 alteration; CR (OR)	k = 3; n = 247 CR-evaluable	OR 1.30 (0.01–156.60)	2.68	80.8	10.40 (2; p = 0.006)	Not shown
DHL-03	DHL/THL-positive vs non-DHL/THL; unadjusted OS (HR)	k = 3; n = 282	HR 1.52 (1.21–1.89)	0	0	0.10 (2; p = 0.951)	HR 0.56–4.08
COO-02	Non-GCB/ABC vs GCB; adjusted PFS (HR)	k = 3; n = 715	HR 1.44 (1.04–2.00)	0	0	0.79 (2; p = 0.675)	HR 0.86–2.43
COO-05	Non-GCB/ABC vs GCB; CR (OR)	k = 3; n = 179 CR-evaluable	OR 1.27 (0.24–6.61)	0	28.9	2.81 (2; p = 0.245)	Not shown

Note: Random-effects models used REML estimation of between-study variance and HKSJ confidence-interval correction for the primary k = 3 pooled estimates. Prediction intervals are shown for HR-based pools only; they were not shown for CR pools because each binary-outcome pool included k = 3 studies with sparse event data. HR >1 indicates worse survival in the biomarker-positive or non-GCB/ABC group; OR >1 indicates higher odds of complete response in the biomarker-positive or non-GCB/ABC group.

Supplementary Table S5.2. Study-level model inputs and random-effects weights

Pool ID	Study [Ref.]	Analysis data	Effect estimate (95% CI)	SE used in model	Weight (%)
TP53-05	Shouval 2022 [25]	CR: 10/29 vs 33/51	OR 0.29 (0.11–0.75)	0.489	37.2
TP53-05	Phuoc 2021 [26]	CR: 5/7 vs 1/8	OR 17.50 (1.22–250.37)	1.358	24.0
TP53-05	Liu 2025a [27]	CR: 21/53 vs 37/99	OR 1.10 (0.56–2.18)	0.349	38.8
DHL-03	Shouval 2022 [25]	N = 153; unadjusted OS	HR 1.44 (0.77–2.70)	0.321	51.3
DHL-03	Bliven 2022 [28]	N = 76; unadjusted OS	HR 1.50 (0.62–3.62)	0.450	26.1
DHL-03	Ghafouri 2021 [29]	N = 53; unadjusted OS	HR 1.73 (0.67–4.46)	0.483	22.7
COO-02	Abid 2025 [30]	N = 344; adjusted PFS	HR 1.40 (1.04–1.89)	0.152	63.4
COO-02	Romano 2023 [31]	N = 64; adjusted PFS	HR 2.01 (0.93–4.35)	0.394	9.5
COO-02	Kwon 2023 [32]	N = 307; adjusted PFS	HR 1.38 (0.87–2.18)	0.233	27.1
COO-05	Zhao 2023 [33]	CR: 11/13 vs 1/3	OR 11.00 (0.65–187.18)	1.446	5.0
COO-05	Romano 2023 [31]	CR: 14/32 vs 11/32	OR 1.49 (0.54–4.08)	0.515	39.4
COO-05	Brinkman 2022 [34]	CR: 15/49 vs 16/50	OR 0.94 (0.40–2.19)	0.433	55.6

Note: SE values correspond to the model scale used for pooling. For HR analyses, SEs were derived from reported 95% CIs. For CR analyses, SEs were derived from the 2×2 complete-response data. Weights are random-effects model weights. Kwon 2023 [32] was inverted from the reported GCB vs non-GCB direction to match the manuscript convention of non-GCB/ABC vs GCB. Romano 2023 [31] contributes to COO-02 and COO-05 because it provided separate adjusted PFS and complete-response data.

Abbreviations: ABC, activated B-cell-like; CI, confidence interval; COO, cell of origin; CR, complete response; df, degrees of freedom; DHL/THL, double-hit/triple-hit lymphoma; GCB, germinal center B-cell-like; HKSJ, Hartung–Knapp–Sidik–Jonkman; HR, hazard ratio; OR, odds ratio; OS, overall survival; PFS, progression-free survival; REML, restricted maximum likelihood; SE, standard error.
