# Supplementary material for: Tumor Genomic Biomarkers as Prognostic Modifiers of Outcomes Following CD19 CAR T-Cell Therapy in Aggressive Large B-Cell Lymphoma: A Systematic Review and Exploratory Meta-Analysis
Source: Genes (Basel). 2026 Jun 30;17(7):752. doi: 10.3390/genes17070752 (PMC13409552; doi:10.3390/genes17070752)
Supplement: Supplementary file 1 [file genes-17-00752-s001.zip › Supplementary_Material S6. Sensitivity analyses for the final pooled meta-analyses (1).pdf]

Supplementary Material S6

Sensitivity analyses for the final pooled meta-analyses

This supplement reports overlapping-cohort and leave-one-out sensitivity analyses for the four final pooled analyses. Each primary pool included three studies; therefore, all sensitivity estimates are based on two-study models and are interpreted descriptively.

For each analysis, one study or flagged cohort was removed and the pooled estimate was recalculated using the remaining two studies. Estimates were calculated on the natural-log scale and back-transformed for presentation as HRs or ORs. The same model specification as the primary analysis was used: random-effects meta-analysis with REML estimation of tau-squared and HKSJ confidence-interval correction. Because only two studies remained after each omission, these estimates should be interpreted as descriptive checks of point-estimate direction and stability, not as formal inferential tests. Study references in Supplementary Tables S6.1 and S6.2 correspond to the numbered reference list in the main manuscript.

Supplementary Table S6.1. Overlapping-cohort sensitivity analyses

Analysis ID	Primary analysis	Cohort omitted {Ref.}	Primary estimate	Sensitivity estimate	Comment
DHL-03	DHL/THL-positive vs non-DHL/THL; unadjusted OS	Ghafouri 2021 [29]	HR 1.52 (95% CI, 1.21-1.89)	HR 1.46 (95% CI, 1.14-1.87)	Direction preserved; estimate decreased slightly.
DHL-03	DHL/THL-positive vs non-DHL/THL; unadjusted OS	Shouval 2022 [25]	HR 1.52 (95% CI, 1.21-1.89)	HR 1.60 (95% CI, 0.65-3.96)	Direction preserved; estimate increased slightly.
COO-02	Non-GCB/ABC vs GCB; adjusted PFS	Abid 2025 [30]	HR 1.44 (95% CI, 1.04-2.00)	HR 1.52 (95% CI, 0.19-12.37)	Direction preserved; CI was very wide.

Note: No separate overlapping-cohort exclusion was required for TP53-05 or COO-05; influence for those pools was assessed by leave-one-out analysis. Overlapping-cohort sensitivity analyses were conceptually distinct from leave-one-out influence analyses. However, because each pooled analysis included only three studies, exclusion of an overlap-flagged cohort was mathematically equivalent to the corresponding leave-one-out iteration.

Supplementary Table S6.2. Leave-one-out influence analyses

Analysis ID/primary estimate	Omitted study [Ref.]	Sensitivity estimate after omission	Summary
DHL-03; HR 1.52 (95% CI, 1.21-1.89)	Shouval 2022 [25]	HR 1.60 (95% CI, 0.65-3.96)	Direction preserved; point estimate increased slightly.
DHL-03; HR 1.52 (95% CI, 1.21-1.89)	Bliven 2022 [28]	HR 1.52 (95% CI, 0.52-4.46)	Point estimate essentially unchanged.
DHL-03; HR 1.52 (95% CI, 1.21-1.89)	Ghafouri 2021 [29]	HR 1.46 (95% CI, 1.14-1.87)	Direction preserved; point estimate decreased slightly.
COO-02; HR 1.44 (95% CI, 1.04-2.00)	Abid 2025 [30]	HR 1.52 (95% CI, 0.19-12.37)	Direction preserved; CI was very wide.
COO-02; HR 1.44 (95% CI, 1.04-2.00)	Romano 2023 [31]	HR 1.39 (95% CI, 1.28-1.52)	Direction preserved; point estimate moved slightly toward 1.0.
COO-02; HR 1.44 (95% CI, 1.04-2.00)	Kwon 2023 [32]	HR 1.47 (95% CI, 0.31-6.90)	Direction preserved; point estimate increased slightly.
TP53-05; OR 1.30 (95% CI, 0.01-156.60)	Shouval 2022]25]	OR 3.21 (95% CI, <0.01-8.85e7)	Direction unchanged; CI was extremely wide.
TP53-05; OR 1.30 (95% CI, 0.01-156.60)	Phuoc 2021 [26]	OR 0.59 (95% CI, <0.01-2.93e3)	Main fragility finding; omission shifted the point estimate below 1.0.
TP53-05; OR 1.30 (95% CI, 0.01-156.60)	Liu 2025a [27]	OR 1.84 (95% CI, <0.01-3.59e11)	Direction unchanged; CI was extremely wide.
COO-05; OR 1.27 (95% CI, 0.24-6.61)	Zhao 2023 [33]	OR 1.13 (95% CI, 0.06-20.18)	Near-null estimate after omitting the small sparse-cell study.
COO-05; OR 1.27 (95% CI, 0.24-6.61)	Romano 2023 [31]	OR 2.18 (95% CI, <0.01-6.16e6)	Estimate moved upward; CI was extremely wide.
COO-05; OR 1.27 (95% CI, 0.24-6.61)	Brinkman 2022 [34]	OR 2.56 (95% CI, <0.01-2.13e5)	Estimate moved upward; CI was extremely wide.

Notes: HR >1 indicates worse survival in the biomarker-positive or non-GCB/ABC group. For CR analyses, OR >1 indicates higher odds of complete response. Values with lower bound reported as <0.01 had lower confidence limits below 0.01 after back-transformation and rounding. Liu 2025a [27] denotes the TP53 CR abstract record; Liu 2025b [41] did not contribute to any pooled analysis.

Abbreviations: ABC, activated B-cell-like; CI, confidence interval; COO, cell of origin; CR, complete response; DHL/THL, double-hit/triple-hit lymphoma; GCB, germinal center B-cell-like; HKSJ, Hartung-Knapp-Sidik-Jonkman; HR, hazard ratio; OR, odds ratio; OS, overall survival; PFS, progression-free survival; REML, restricted maximum likelihood.
