# Supplementary material for: Tumor Genomic Biomarkers as Prognostic Modifiers of Outcomes Following CD19 CAR T-Cell Therapy in Aggressive Large B-Cell Lymphoma: A Systematic Review and Exploratory Meta-Analysis
Source: Genes (Basel). 2026 Jun 30;17(7):752. doi: 10.3390/genes17070752 (PMC13409552; doi:10.3390/genes17070752)
Supplement: Supplementary file 1 [file genes-17-00752-s001.zip › Supplementary_Material S7. Per-study contribution tables for pooled analyses.pdf]

Supplementary Material S7

Per-study contribution tables for pooled analyses

This supplement identifies the source studies contributing to each final pooled analysis. Detailed log-scale model inputs, standard errors, and weights are reported in Supplementary Material S5. Study references in Supplementary Tables S7.1, S7.2, S7.3, and S7.4 correspond to the numbered reference list in the main manuscript.

Supplementary Table S7.1. TP53-05: TP53 alteration and complete response

Study/report	Source and setting	Extracted data	Model input used	Contribution note
Shouval 2022 [25]	Full-text article; single-center real-world cohort (MSKCC); 3L+ CAR T-cell.	CR: 10/29 vs 33/51.	OR 0.287 (0.110–0.748).	Contributed TP53-altered vs TP53 wild-type CR data. Response denominator differed from the full sequenced cohort.
Phuoc 2021 [26]	Conference abstract; retrospective real-world cohort; 3L+ CAR T-cell.	CR: 5/7 vs 1/8.	OR 17.500 (1.223–250.369).	Contributed binary CR data; small cohort and sparse cells drove influence in sensitivity analysis.
Liu 2025a [27]	Conference abstract; retrospective real-world cohort; China.	CR: 21/53 vs 37/99.	OR 1.100 (0.555–2.181).	Contributed binary CR data; no Cox HR with 95% CI was available for survival pooling.

Supplementary Table S7.2. DHL-03: DHL/THL status and unadjusted overall survival

Study/report	Source and setting	Extracted data	Model input used	Contribution note
Shouval 2022 [25]	Full-text article; single-center real-world cohort (MSKCC); 3L+ CAR T-cell.	Unadjusted OS HR for DHL/THL-positive vs non-DHL/THL disease.	HR 1.440 (0.768–2.701).	Contributed unadjusted OS estimate. PFS HR for this contrast was not reported.
Bliven 2022 [28]	Full-text article; EHR-derived real-world cohort (Flatiron); 3L+ CAR T-cell.	Unadjusted OS HR for DHL/THL-positive vs non-DHL/THL disease.	HR 1.500 (0.621–3.623).	Contributed unadjusted OS estimate. DHL/THL denominators within the CAR T-cell subgroup were not reported in the source text.
Ghafouri 2021 [29]	Full-text article; single-institution real-world cohort (UCLA); 3L+ axi-cel.	Unadjusted OS HR for DHL/THL-positive vs non-DHL/THL disease.	HR 1.730 (0.672–4.456).	Full publication superseded the earlier abstract precursor and contributed the final UCLA cohort estimate.

Supplementary Table S7.3. COO-02: cell of origin and adjusted progression-free survival

Study/report	Source and setting	Extracted data	Model input used	Contribution note
Abid 2025 [30]	Conference abstract; single-center real-world cohort (MD Anderson); 3L+ CAR T-cell.	Adjusted PFS HR for non-GCB vs GCB COO.	HR 1.400 (1.039–1.887).	Contributed adjusted PFS estimate; evaluated in overlapping-cohort sensitivity analysis.
Romano 2023 [31]	Conference abstract; ZUMA-7 second-line axi-cel subgroup.	Adjusted PFS HR for non-GCB vs GCB COO.	HR 2.010 (0.929–4.347).	Contributed adjusted PFS estimate and also contributed CR data to COO-05.
Kwon 2023 [32]	Full-text article; Spanish multicenter real-world registry (GETH-TC/GELTAMO); 3L+ CAR T.	Adjusted PFS HR; reported direction was GCB vs non-GCB.	HR 1.380 (0.874–2.179).	Estimate was inverted to match the manuscript convention of non-GCB/ABC vs GCB.

Supplementary Table S7.4. COO-05: cell of origin and complete response

Study/report	Source and setting	Extracted data	Model input used	Contribution note
Zhao 2023 [33]	Full-text article; single-center molecular cohort; China; mixed-line CAR T-cell.	CR: 11/13 vs 1/3.	OR 11.000 (0.646–187.176).	Contributed binary CR data; small GCB comparator group led to sparse-cell imprecision.
Romano 2023 [31]	Conference abstract; ZUMA-7 second-line axi-cel subgroup.	CR: 14/32 vs 11/32.	OR 1.485 (0.541–4.077).	Contributed binary CR data and also contributed adjusted PFS data to COO-02.
Brinkman 2022 [34]	Conference abstract; retrospective real-world cohort; 3L+ CAR T-cell.	CR: 15/49 vs 16/50.	OR 0.937 (0.401–2.193).	Contributed binary CR data. Time-to-event outcomes were reported only as Kaplan-Meier/log-rank results and were not pooled.

Notes: For CR analyses, values are shown as biomarker-positive events/total versus biomarker-negative events/total. HR >1 indicates worse survival in the biomarker-positive or non-GCB/ABC group; OR >1 indicates higher odds of complete response. Study labels correspond to Supplementary Table S3.1.

Abbreviations: ABC, activated B-cell-like; axi-cel, axicabtagene ciloleucel; CAR T-cell, chimeric antigen receptor T-cell therapy; COO, cell of origin; CR, complete response; DHL/THL, double-hit/triple-hit lymphoma; EHR, electronic health record; GCB, germinal center B-cell-like; HR, hazard ratio; MSKCC, Memorial Sloan Kettering Cancer Center; OR, odds ratio; OS, overall survival; PFS, progression-free survival; UCLA, University of California, Los Angeles.
